# Supplementary material for: Identity and functions of monoaminergic neurons in the predatory nematode Pristionchus pacificus reveal nervous system conservation and divergence
Source: bioRxiv. 2025 Oct 16:2025.10.16.682888. Preprint. [Version 1] doi: 10.1101/2025.10.16.682888 (PMC12632918; doi:10.1101/2025.10.16.682888)
Supplement: 1 [file NIHPP2025.10.16.682888v1-supplement-1.pdf]

bioRxiv preprint doi: https://doi.org/10.1101/2025.10.16.682888; this version posted October 16, 2025. The copyright holder for this preprint (which was not certified by peer review) is the author/funder, who has granted bioRxiv a license to display the preprint in perpetuity. It is made available under aCC-BY 4.0 International license.

Supplemental Materials and Methods

dat-1 and *cha-1* alleles: These indel alleles were generated during screens intended to epitope-tag the genes. Injection mixes were prepared using the method described in (Dokshin et al., 2018) for *C. elegans,* with the addition of lipofectamine (Adams et al., 2019): Cas9 - 0.5 µl at 10 µg/µl, tracrRNA – 5 µl at 0.4 µg/µl, 2.8 µl at 100 µM. This mixture was incubated for 10 min at 37°C. Then 0.6 µl Lipofectamine RNAiMax reagent (final conc 3%, Thermofisher 1377803) and 1 µl single stranded repair template at 100 µM were added, plus 12.1 µl nuclease free water to an injection mix final volume of 20 µl. For the *cha-1* screen, 16 young adult hermaphrodites (worms with ≤2 eggs) were injected and placed on individual plates; these parents were removed after ~24 hrs of laying eggs. Three days later, ≤ 20 F1s were singled from each parental plate (some P0's had fewer than 20 progeny). Two or more days later, after the adult had many progeny eggs and larvae, screening by PCR was performed using a modified 'single worm' PCR method (Williams et al., 1992). The F1 adult plus several larvae and eggs were picked into 2 µl PCR / Proteinase K lysis buffer (0.5 mg/ml proteinase K final concentration, prepared fresh) in the lid of a PCR tube. Lysis buffer with worms was spun down, frozen in liquid nitrogen, then immediately thawed in a water bath at 37-65°C, then repeated; the rapid freeze-thaw cycle was performed a total of 3 times. PCR tubes were put in a Thermocycler, digested at 65 C for 60 min, heat-inactivated at 95 C for 15 min, then cooled to 4°C, and within ~1 hr, PCR mix with appropriate primers was added, and PCR performed with primers flanking the expected cut & insertion site. Clone plates yielding PCR bands with increased sizes consistent with repair template insertion were selected for further subcloning to isolate homozygous mutants. Among the homozygotes isolated were indel mutants. For the *cha-1* screen, 271 F1 clones were screened, yielding one proper 2x FLAG epitope C-terminal insertion and 3 indel mutants. One mutant was apparently homozygous larval lethal, with heterozygotes throwing ~ ¼ early larval arrest coilers (a phenotype like seen in *C. elegans cha-1* null homozygotes); this strain was eventually lost. For the *dat-1* screen, 15 worms were injected; parents removed after ~48 hrs. Because of a need to delay F1 singling, those plates were then put at 15°C for 4 days, at which time ~ 270 F1s were singled and subsequently grown at standard temperature. 158 clones were screened by PCR; from an initial screen of 94, ~17% appeared to have a genomic alteration (from one 'jackpot' P_0 plate, 50% of F1's showed genomic alteration). Therefore, the remainder of clones

bioRxiv preprint doi: https://doi.org/10.1101/2025.10.16.682888; this version posted October 16, 2025. The copyright holder for this preprint (which was not certified by peer review) is the author/funder, who has granted bioRxiv a license to display the preprint in perpetuity. It is made available under aCC-BY 4.0 International license.

were not followed up. Subcloning and rescreening resulted in the isolation of 5 independent strains with the proper N-terminal HA tag insertion, 1 strain with an indel mutation and partial tag insertion, and 3 strains with indel mutations, all resulting in likely nulls.

bioRxiv preprint doi: https://doi.org/10.1101/2025.10.16.682888; this version posted October 16, 2025. The copyright holder for this preprint (which was not certified by peer review) is the author/funder, who has granted bioRxiv a license to display the preprint in perpetuity. It is made available under aCC-BY 4.0 International license.

Supplemental Figures

Suppl Fig S1

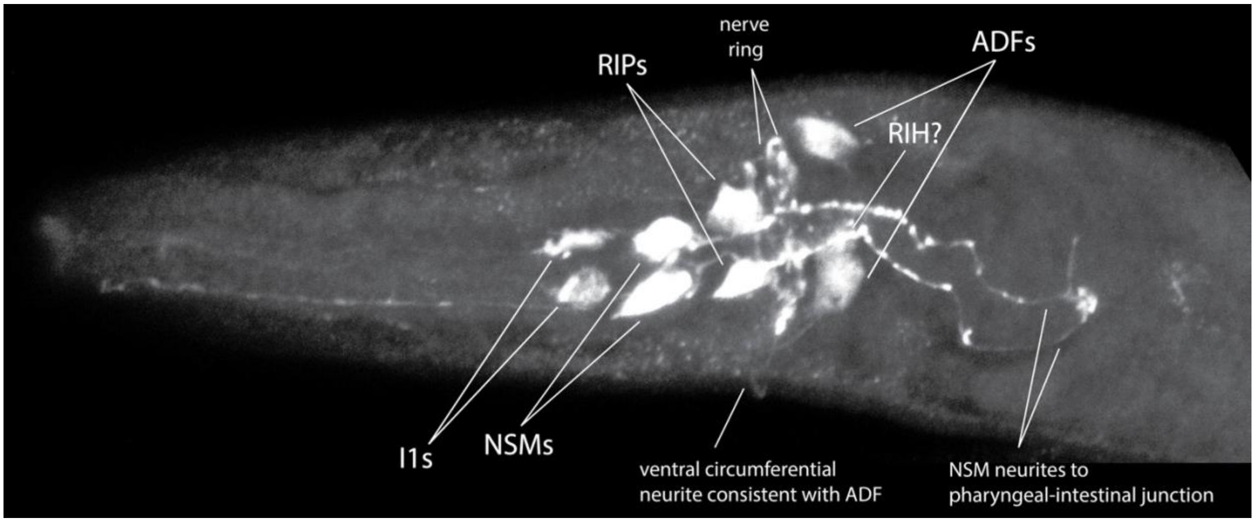

Suppl Fig S1 – Anti-5HT staining of wildtype *P. pacificus*, larval head showing all serotonin-IR head neurons as in Figure 1 adult, Max IP, anterior to the left, ventral view. In the pharynx: I1s, NSMs; anterior ganglion in front of the nerve ring: RIPs; posterior to the nerve ring: ADFs and unpaired possible RIH. Because the pharynx is not kinked as it Figure 5, the unpaired neuron is shown clearly to be posterior to the nerve ring.

Suppl Fig S2

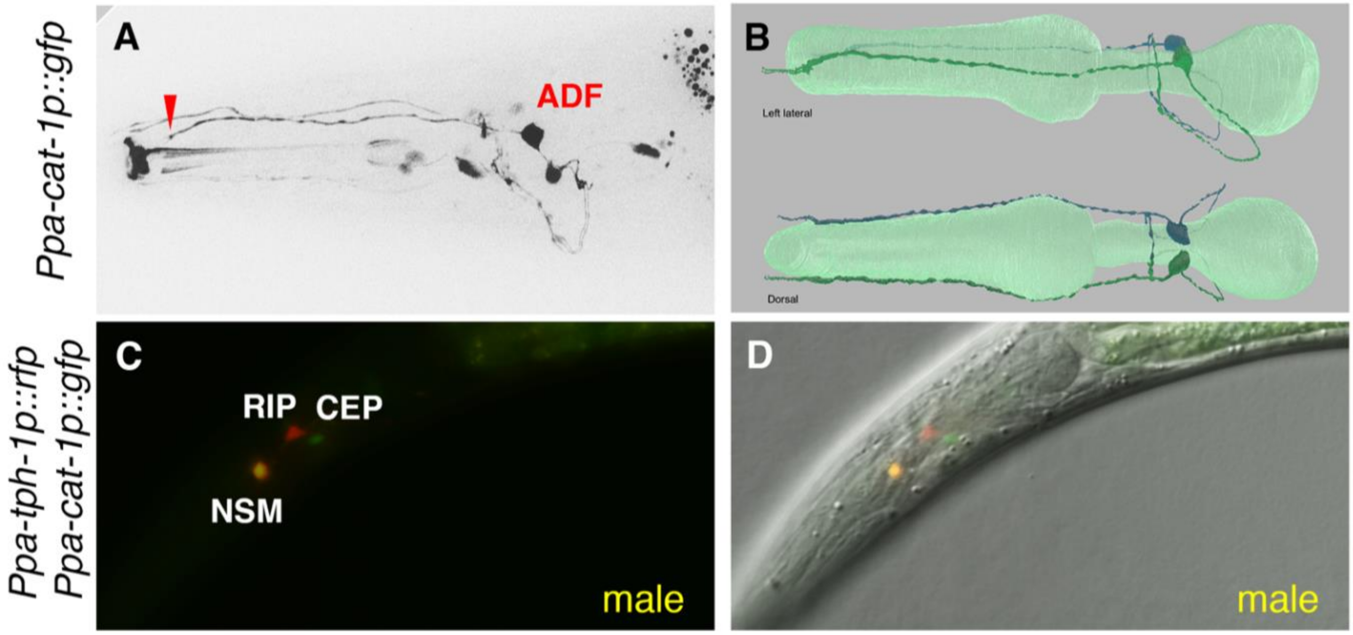

Suppl Fig S2 – Additional reporter expression in aminergic neurons in *P. pacificus.* (A) *cat-1p::gfp* expression in the amphid neuron homolog ADF(AM9) with double ciliated ending (arrowhead). Anterior is to the left. (B) 3D-rendering of the ADF neuron pair in lateral and dorsal orientations. (C-D) F_1 hermaphrodite adult showing overlap of *cat-1p::gfp* and *tph-1p::rfp* reporter expression in the NSM neuron.

bioRxiv preprint doi: https://doi.org/10.1101/2025.10.16.682888; this version posted October 16, 2025. The copyright holder for this preprint (which was not certified by peer review) is the author/funder, who has granted bioRxiv a license to display the preprint in perpetuity. It is made available under aCC-BY 4.0 International license.

Suppl Fig S3

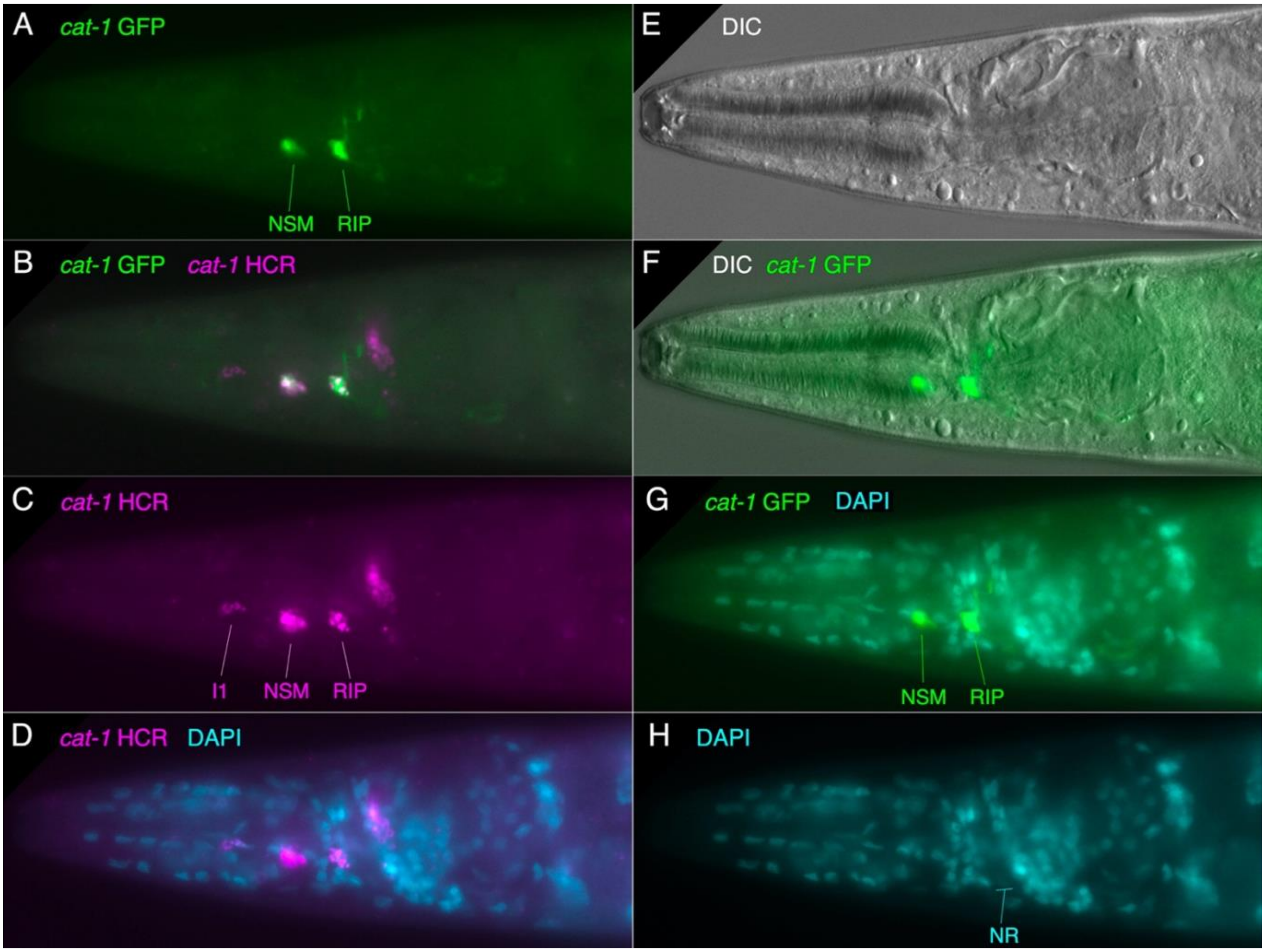

Suppl Fig S3 – Expression of *cat-1* transcripts via HCR in head in *cat-1*::GFP strain showing RIP neuron in anterior ganglion with neurite to dorsal nerve ring. Anterior to the left, ventro-lateral view of head. All images are the same focal plane, except (E), slightly different to show pharynx outline better. (A) Mosaic expression of *cat-1::GFP* reporter in NSM and RIP on one side of the head. Note the characteristic morphology of RIP neurites. (B) Colocalization of *cat-1* transcripts with *cat-1::GFP* in NSM and RIP. (C) *cat-1* transcripts (magenta). (D) *cat-1* transcripts and DAPI showing RIP in anterior ganglion anterior to nerve ring (nucleus-free region, marked in H). (E) DIC alone (F) DIC with *cat-1::GFP*. (G) *cat-1::GFP* with DAPI showing RIP ending in dorsal nerve ring. (H) DAPI showing nuclei and location of nerve ring (NR).

bioRxiv preprint doi: https://doi.org/10.1101/2025.10.16.682888; this version posted October 16, 2025. The copyright holder for this preprint (which was not certified by peer review) is the author/funder, who has granted bioRxiv a license to display the preprint in perpetuity. It is made available under aCC-BY 4.0 International license.

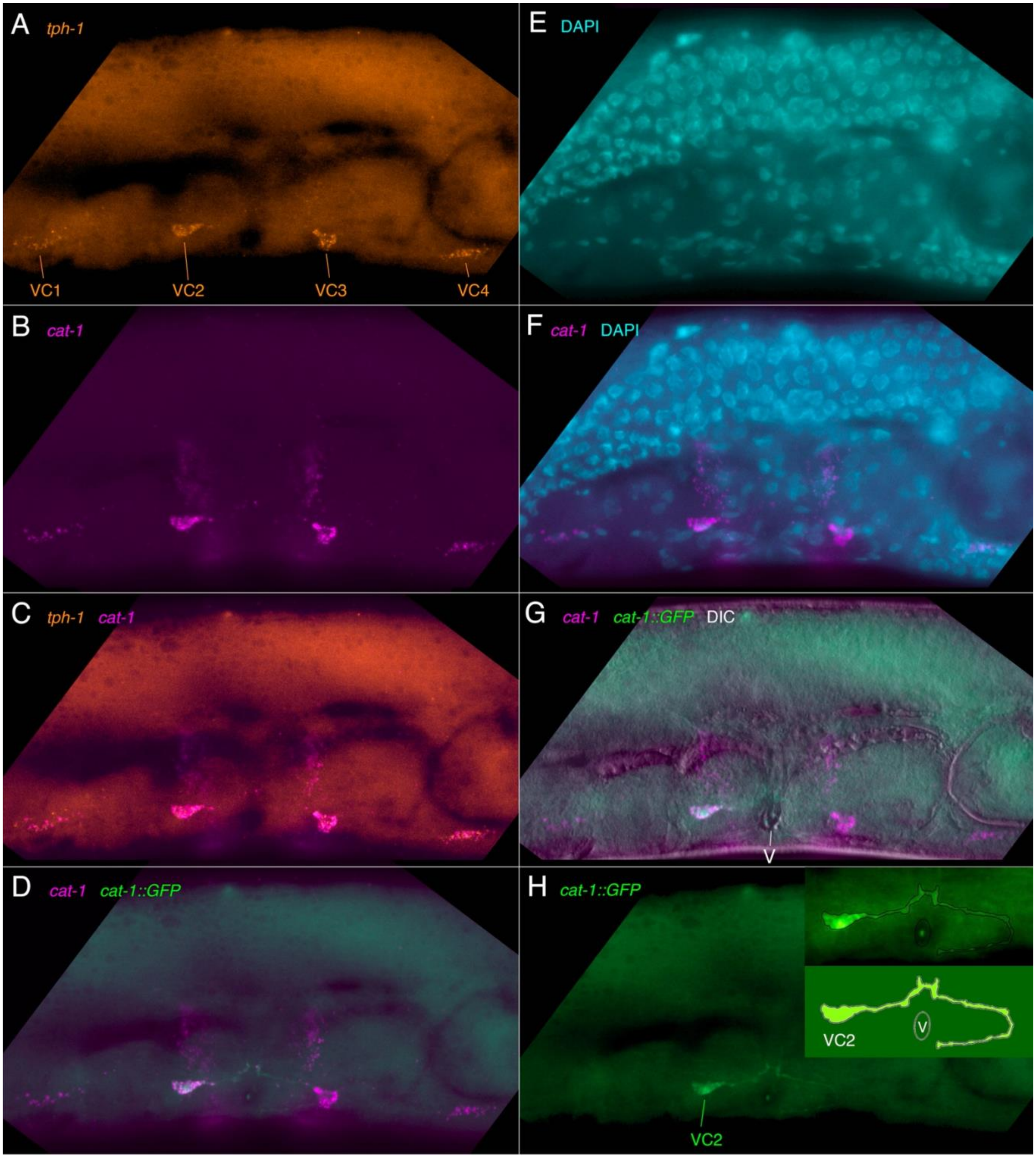

Suppl Fig S4 – Coexpression of *tph-1* and *cat-1* transcripts in vulval region in *cat-1*::GFP strain. Anterior to the left, ventral down, all images of the same focal plane. (A) *tph-1* transcripts expressed in VC1-4 in the VNC (B) *cat-1* transcripts expressed in VC1-4 and in vulval cells in ventrolateral body wall. (C) *tph-1* and *cat-1* transcripts colocalized in VC1-4. (D) *cat-1* transcripts colocalized with *cat-1*::GFP reporter fluorescence of a single labeled VC neuron, VC2. (E) DAPI staining of vulval region, showing compact nuclei of VNC. (F) *cat-1* transcripts colocalized with DAPI-stained VNC nuclei. (G) Including DIC shows the location of vulval pore between VC2 & VC3 (marked with 'V'). (H) *cat-1*::GFP alone, showing VC2 neuron. Interestingly, the neurite crosses to the other side of the vulval pore and grows back anteriorly with varicosities. Inset: schematic outline of VC2 major branches. (Note that fine branches may not be seen.)

bioRxiv preprint doi: https://doi.org/10.1101/2025.10.16.682888; this version posted October 16, 2025. The copyright holder for this preprint (which was not certified by peer review) is the author/funder, who has granted bioRxiv a license to display the preprint in perpetuity. It is made available under aCC-BY 4.0 International license.

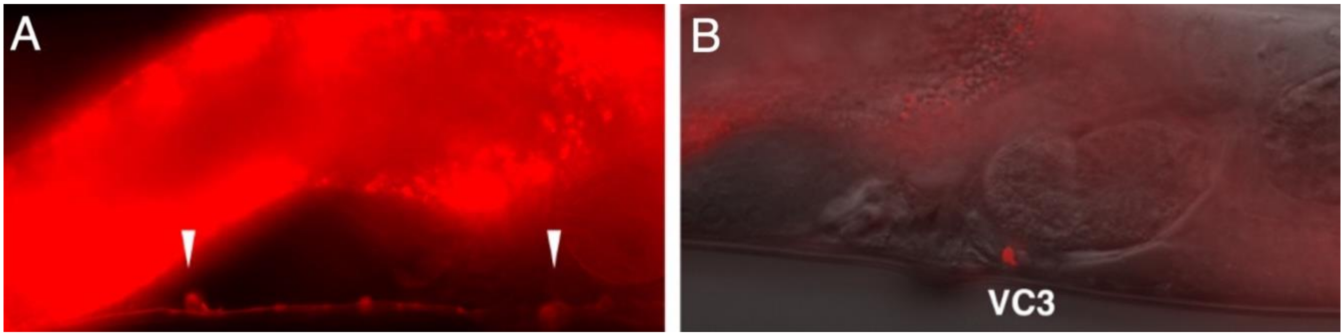

Suppl Fig S5 – Cells expressing the *tph-1p::rfp* reporter in the mid-body of *P. pacificus* (A, B) VC neurons in the VNC express the *tph-1p::rfp* reporter. (A) Arrowheads indicate somas in ventral nerve cord near the vulva; neurites are also apparent. (B) VC3 neuron soma just posterior to the vulva, including DIC.

Suppl Fig S6 – Serotonin-IR is unchanged in *mod-5* mutants

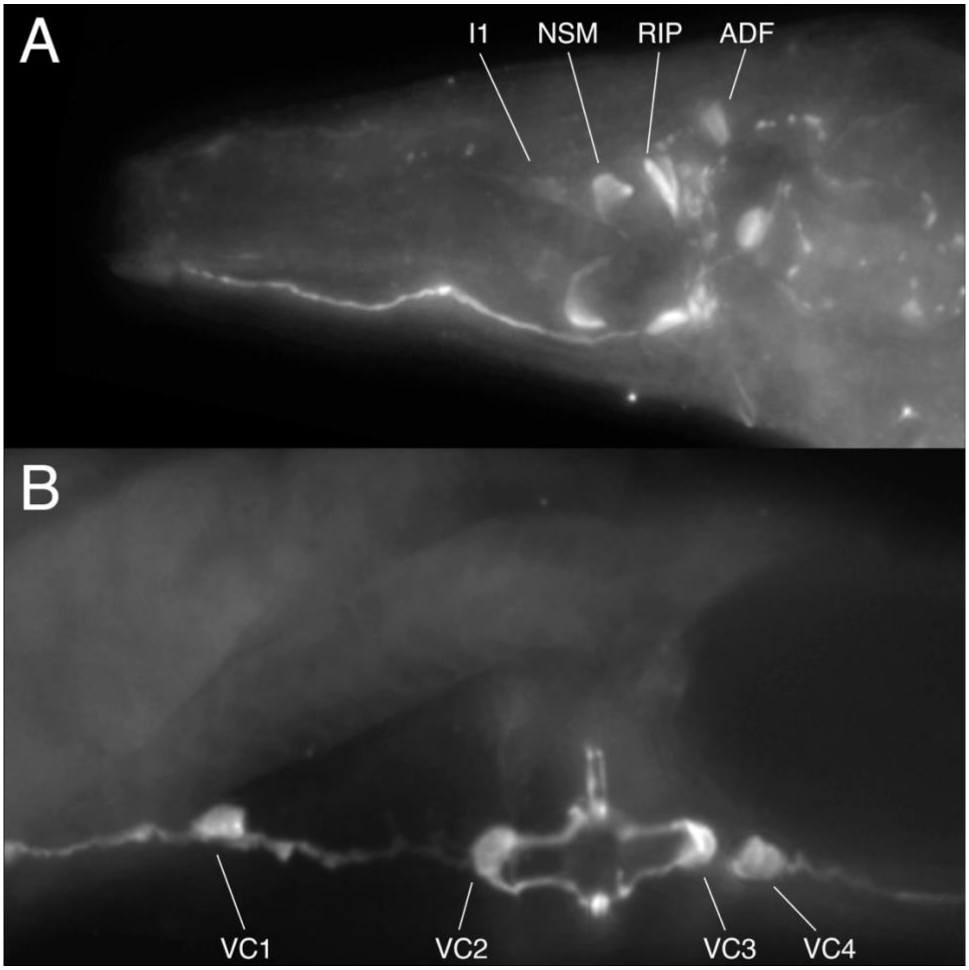

Suppl Fig S6 – Serotonin-IR is unchanged in *mod-5* mutants

Anti-serotonin staining in *mod-5(tu587)* mutant. Anterior to the left. The same result was seen with the other allele, *mod-5(tu586)*. (A) Adult hermaphrodite head, MaxIP of several focal planes, approximately dorsal-ventral view. All serotonin-IR cells (I1s, NSMs, RIPs, and ADFs) are seen, as in wildtype. Staining of the unpaired ventral serotonin-IR neuron, which is weak and unreliable, was not seen in wildtype worms in these experiments, so this cell might still be uptake-dependent. (B) Adult midbody vulval region, ventro-lateral view. VC neurons stain as in wildtype. We observed the same results for worms treated with SERT blockers fluoxetine or imipramine at various concentrations; no cells were affected except at the highest concentration used, which eliminated or greatly reduced serotonin staining uniformly in all cells, a likely non-specific effect since these drugs can bind other targets beside SERT.

bioRxiv preprint doi: https://doi.org/10.1101/2025.10.16.682888; this version posted October 16, 2025. The copyright holder for this preprint (which was not certified by peer review) is the author/funder, who has granted bioRxiv a license to display the preprint in perpetuity. It is made available under aCC-BY 4.0 International license.

Suppl Fig S7 – Serotonergic neurons in the head express *mod-5/SERT*

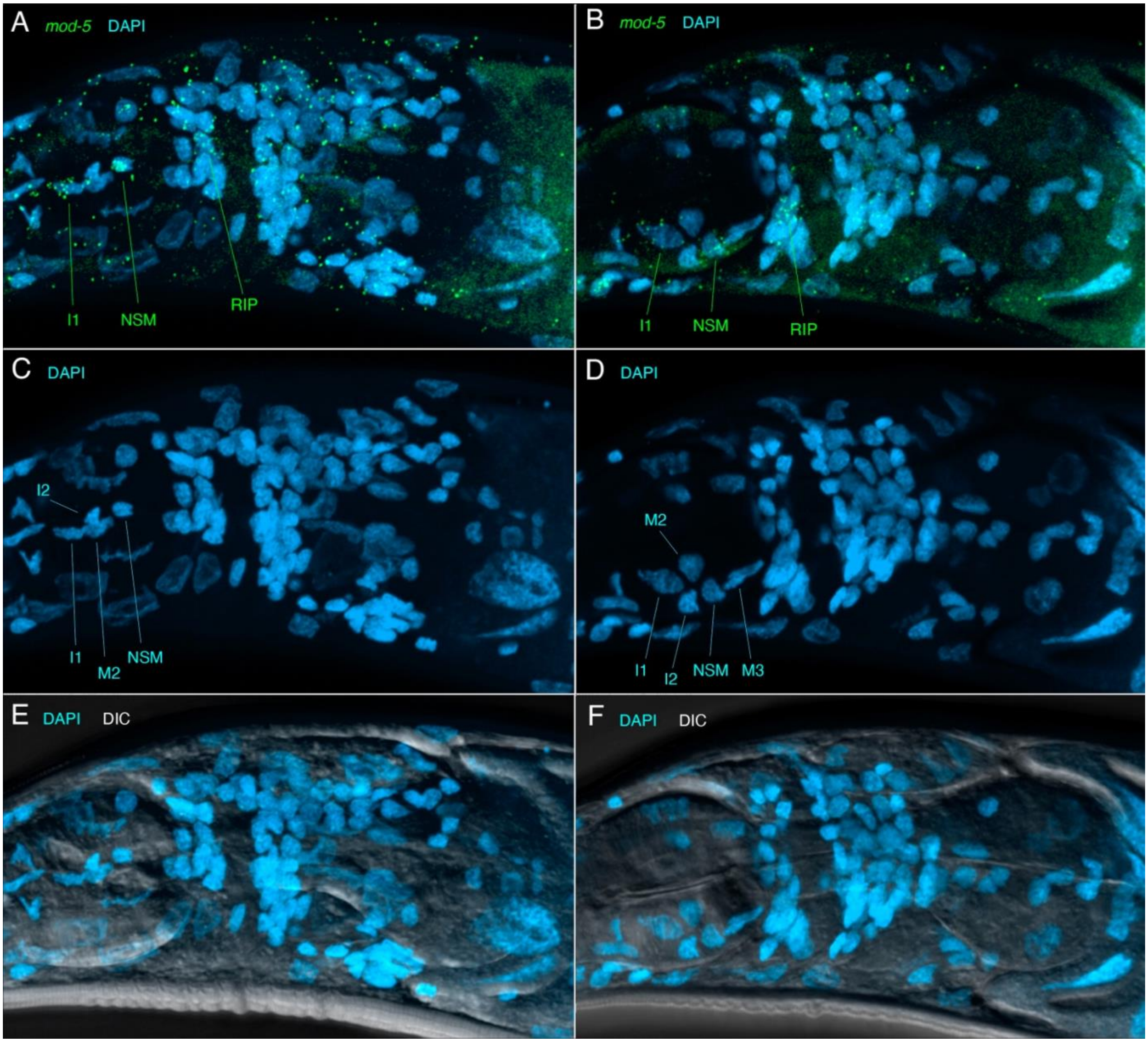

Suppl Fig S7 – Serotonergic neurons in the head express *mod-5/SERT* transcripts
Anterior to the left, ventrolateral views with ventral approximately down. MaxIPs of a few focal planes on left (A,C,E) and right sides (B, D, F) of head. (A) Expression of *mod-5* transcripts (green) in identified pharyngeal (I1, NSM) and anterior ganglion (RIP) serotonin neurons, left side, with DAPI (cyan). (B) Same on the right side. (C) DAPI alone, same as A, with other adjacent DAPI-stained pharyngeal neurons identified. (D) Same on the right side. (E) Including DIC to show outlines of pharyngeal bulbs. (F) Same on the right side.

bioRxiv preprint doi: https://doi.org/10.1101/2025.10.16.682888; this version posted October 16, 2025. The copyright holder for this preprint (which was not certified by peer review) is the author/funder, who has granted bioRxiv a license to display the preprint in perpetuity. It is made available under aCC-BY 4.0 International license.

Suppl Fig S8 – Head serotonergic and other neurons in the head sometimes express *mod-5/SERT*

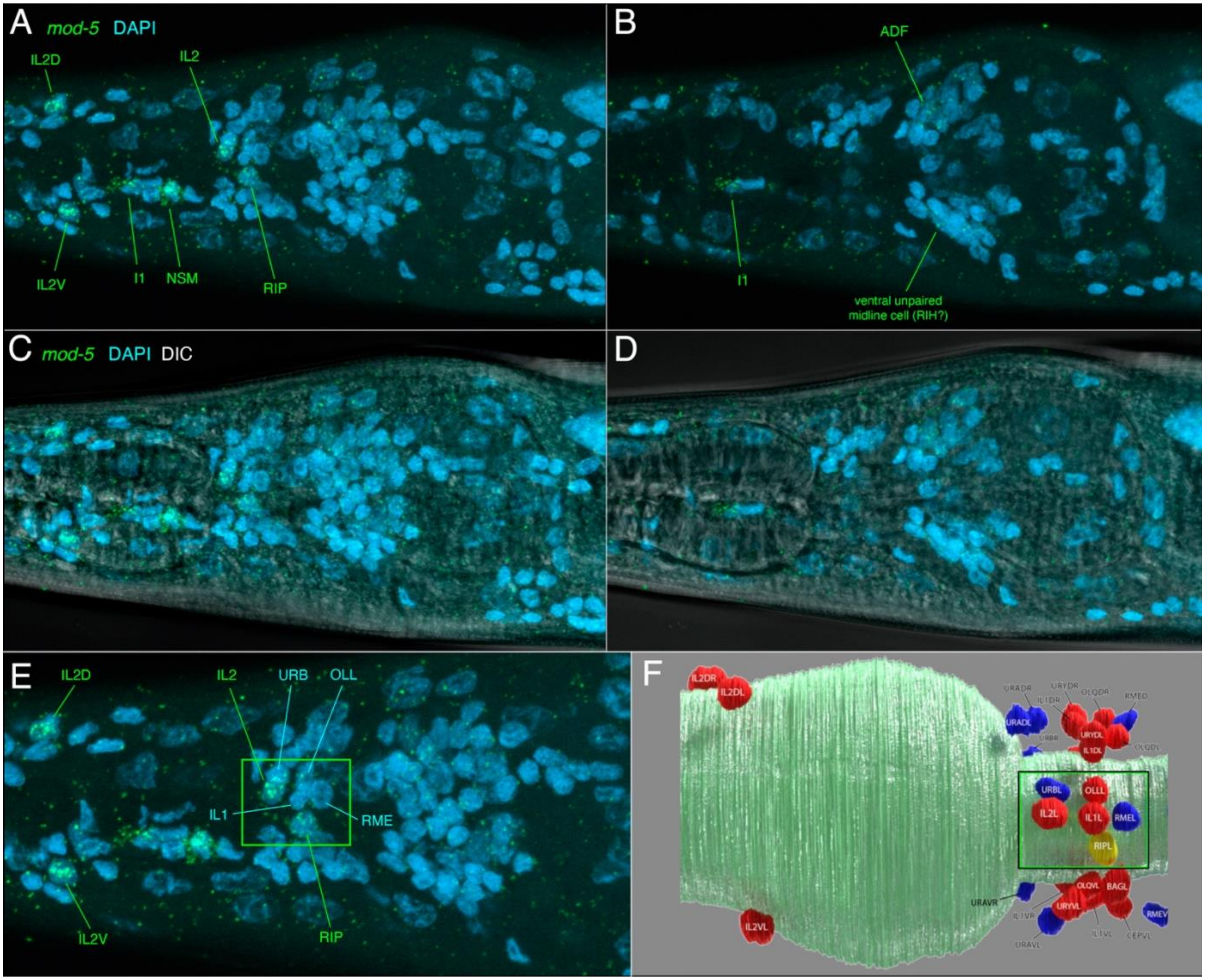

Suppl Fig S8 – Head serotonergic and other neurons in the head sometimes express *mod-5/SERT*
Anterior to the left, ventral down, all images. MaxIPs of the same few focal planes (A, C, E), or a nearby set of focal planes (B, D). (A) Expression of *mod-5* transcripts (green) in identified pharyngeal (I1, NSM) and anterior ganglion (RIP) serotonin neurons, plus IL2 neurons (D, V, lateral), with DAPI (cyan). (B) Nearby focal planes showing *mod-5* transcripts in serotonin neurons posterior to the nerve ring, ADF and possibly RIH. (C, D) Including DIC to show outlines of pharyngeal bulbs. (E) Closeup of anterior ganglion, lateral neurons (green box). Several adjacent DAPI-stained neuronal nuclei are identified by position. (F) Map of identified anterior ganglion neuronal nuclei, left side lateral view (boxed as in E), 3D rendering from EM reconstruction (Cook et al., 2025).

bioRxiv preprint doi: https://doi.org/10.1101/2025.10.16.682888; this version posted October 16, 2025. The copyright holder for this preprint (which was not certified by peer review) is the author/funder, who has granted bioRxiv a license to display the preprint in perpetuity. It is made available under aCC-BY 4.0 International license.

Suppl Fig S9 – Vulval-proximal VC neurons express *mod-5/SERT*

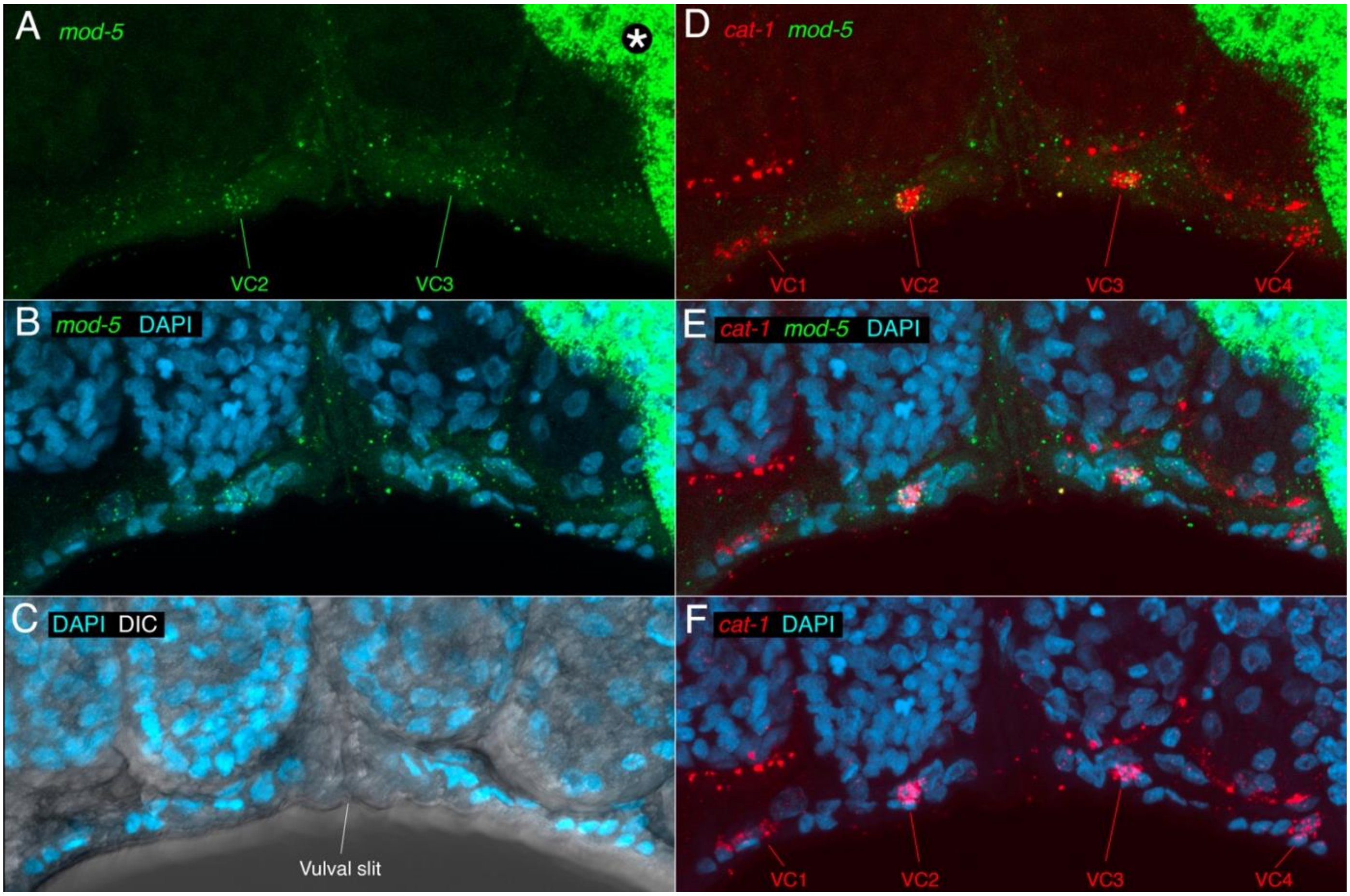

Suppl Fig S9 – Vulval-proximal VC neurons express *mod-5/SERT*
Closeup of vulval region; anterior to the left, ventral down. Images are MaxIPs of several focal planes. (A) *mod-5* transcripts expressed in VC2 and VC3 (proximal VCs) in the VNC, but not obviously in distal VCs (VC1, VC4). Asterisk indicates intestine, which displays high background and autofluorescence in this preparation. (B) *mod-5* transcripts are associated with compact VNC nuclei shown with DAPI staining. (C) DIC reveals location of vulval opening. Four embryos in the uterus are seen above the VNC. (D) *cat-1* transcripts are expressed in VC1-4, but colocalized with *mod-5* only in proximal VCs. (E) DAPI staining of vulval region, showing compact nuclei of VNC. (F) *cat-1* transcripts colocalized with DAPI-stained VNC nuclei.

bioRxiv preprint doi: https://doi.org/10.1101/2025.10.16.682888; this version posted October 16, 2025. The copyright holder for this preprint (which was not certified by peer review) is the author/funder, who has granted bioRxiv a license to display the preprint in perpetuity. It is made available under aCC-BY 4.0 International license.

Suppl Fig S10 – Expression of cat-2 transcripts in identified dopaminergic neurons

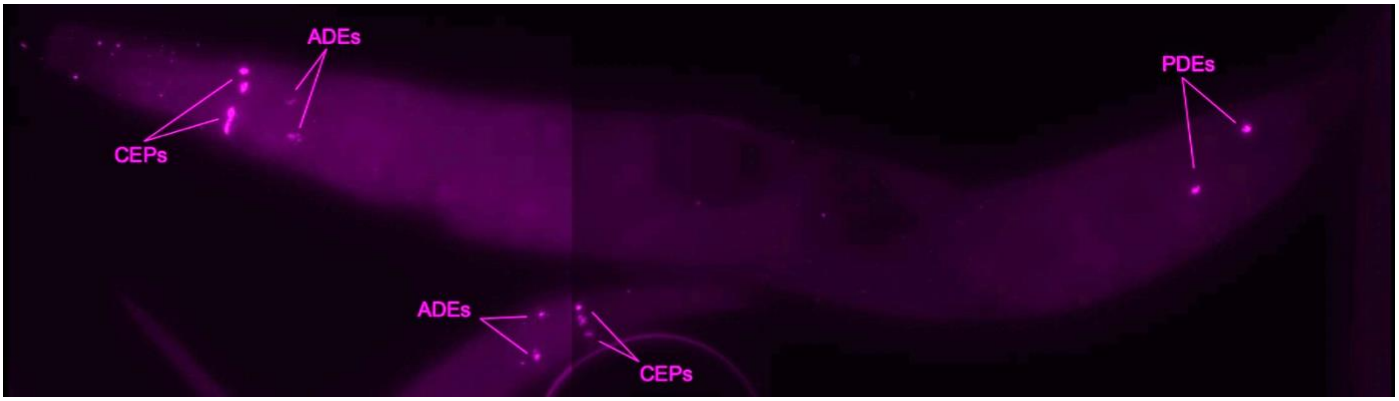

Suppl Fig S10 – Expression of cat-2 transcripts in identified dopaminergic neurons
Whole adult (anterior to the left) and larval head (anterior to right) expression of *cat-2* transcripts (magenta), MaxIP. Head neurons CEPs and ADEs are seen both in the adult head (top) and larval head (below). The posterior body shows PDE neurons on left and right sides.

Suppl Fig S11

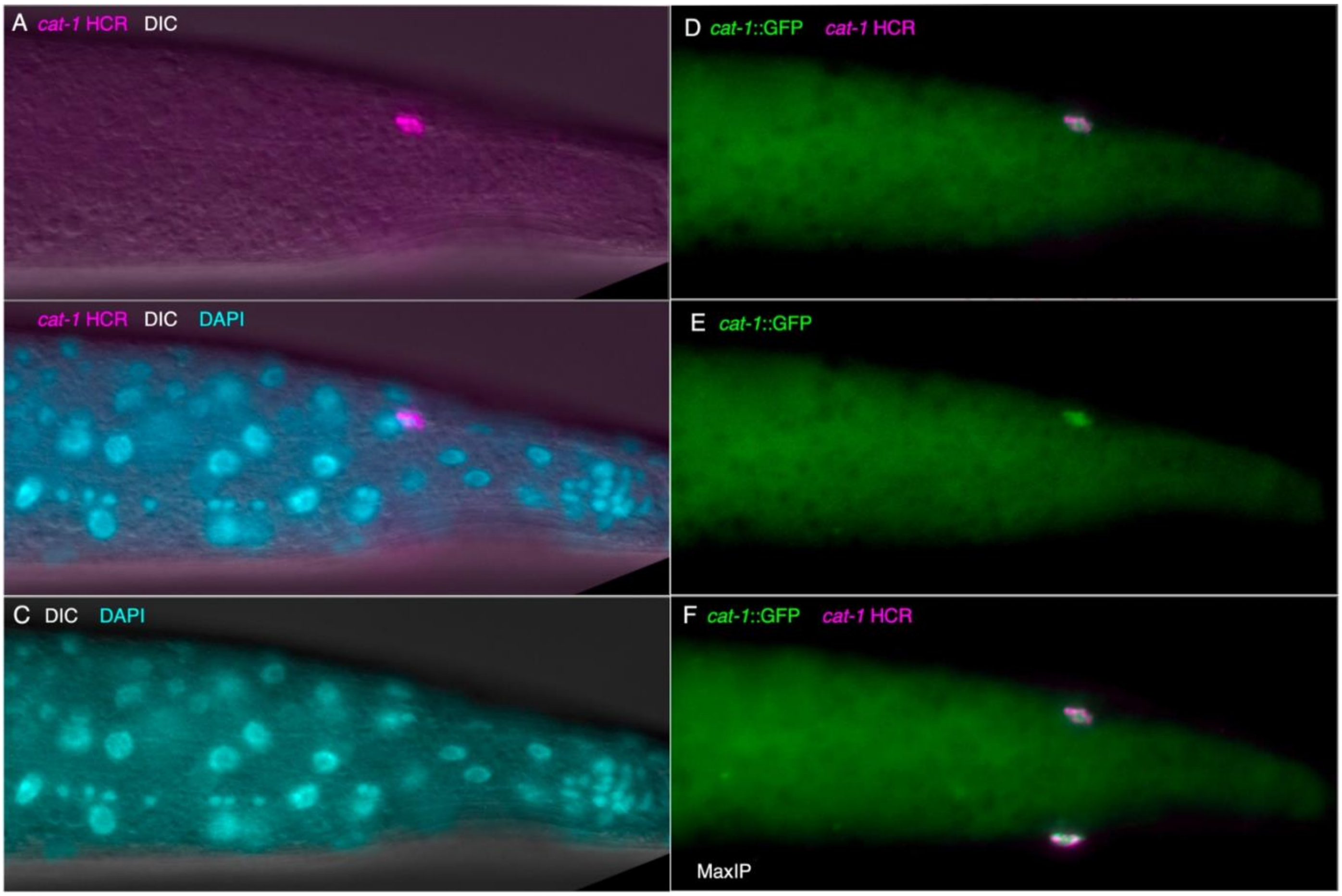

Suppl Fig S11 – Colocalization of *cat-1 transcripts* and *cat-1*::GFP reporter in posterior body dopaminergic neurons (PDEs). Anterior is to the left in all panels. (A-E) Single focal plane. (A) *cat-1* HCR fluorescence (magenta) and DIC. (B) As in A, with DAPI. (C) DIC and DAPI, which shows a small, compact nucleus associated with the *cat-1* HCR signal. (D) *cat-1*::GFP (green) and *cat-1* transcripts. (E) *cat-1*::GFP alone (F) MaxIP of several focal planes to show colocalization in PDE neurons on both sides of the body wall.

bioRxiv preprint doi: https://doi.org/10.1101/2025.10.16.682888; this version posted October 16, 2025. The copyright holder for this preprint (which was not certified by peer review) is the author/funder, who has granted bioRxiv a license to display the preprint in perpetuity. It is made available under aCC-BY 4.0 International license.

Suppl Fig S12 – Co-expression of *cat-2* and *cat-1* transcripts in PDE neurons

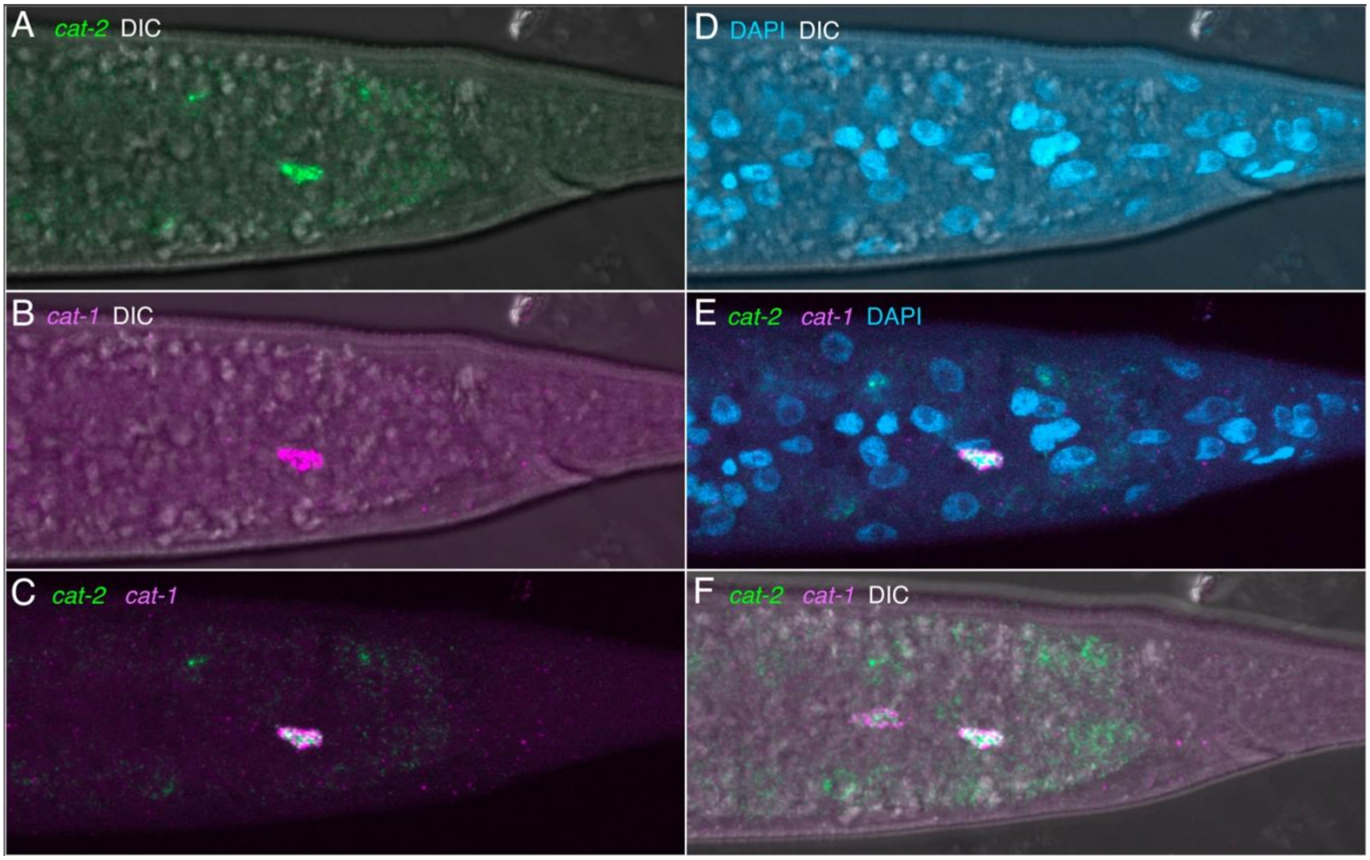

Suppl Fig S12 – Co-expression of *cat-2* and *cat-1* transcripts in PDE neurons. Anterior is to the left in all panels. (A-E) Right side of posterior body, MaxIP of same set of focal planes. (A) *cat-2* HCR fluorescence (green) and DIC. (B) *cat-1* HCR fluorescence (magenta) and DIC. (C) Colocalization of *cat-2* and *cat-1* transcripts in PDE neuron. (D) DAPI and DIC (E) *cat-2* and *cat-1* transcripts are associated with compact PDER nucleus associated with the *cat-1* HCR signal. (F) MaxIP including left side focal planes to show colocalization in PDE neurons on both sides of the body.

Suppl Fig S13

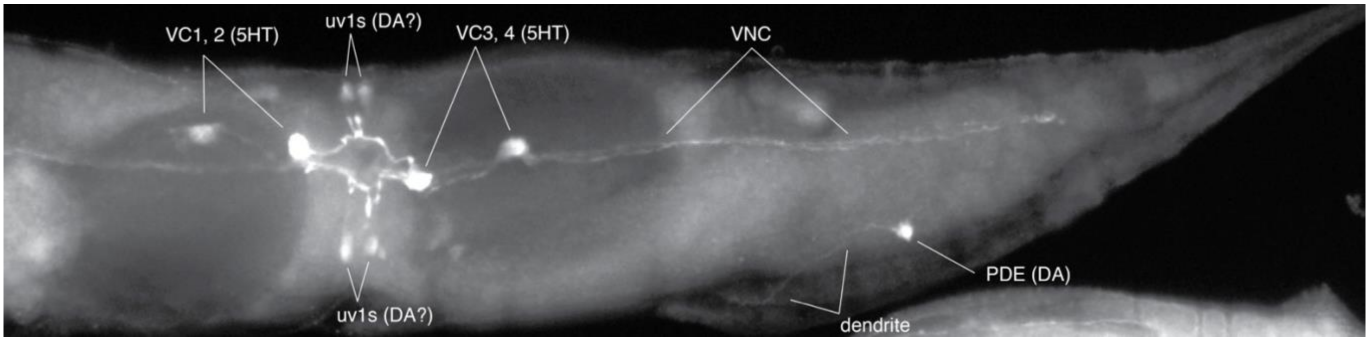

Suppl Fig S13 – Monoaminergic cells in the central and posterior body of *P. pacificus*
Anterior is to the left. Serotonergic and presumptive dopaminergic cells and in the central and posterior body of adult hermaphrodite *P. pacificus* revealed by 5-HTP-induced serotonin immunoreactivity; animal incubated with 5-HTP and subsequently stained with anti-serotonin. Dopaminergic neurons take up 5-HTP and convert it to serotonin. In the ventral nerve cord, VC motor neurons stain for serotonin (and are also seen *without* 5-HTP treatment). On either side of the vulva in the lateral body wall are teardrop-shaped cells that are likely homologs of *C. elegans* uterine vulva uv1 cells. In the mid-posterior lateral body wall is the dopaminergic PDE mechanosensory neuron, which extends a long dendrite anteriorly and laterally (with a slight turn dorsally at the end, in contrast to that seen in other free-living nematodes in which a much shorter dendrite extends dorsally from the soma (Rivard et al., 2010). The PDE dendrite is seen more clearly in Fig. 6E-F, showing *cat-1p::gfp* expression.

bioRxiv preprint doi: https://doi.org/10.1101/2025.10.16.682888; this version posted October 16, 2025. The copyright holder for this preprint (which was not certified by peer review) is the author/funder, who has granted bioRxiv a license to display the preprint in perpetuity. It is made available under aCC-BY 4.0 International license.

Suppl Fig S14

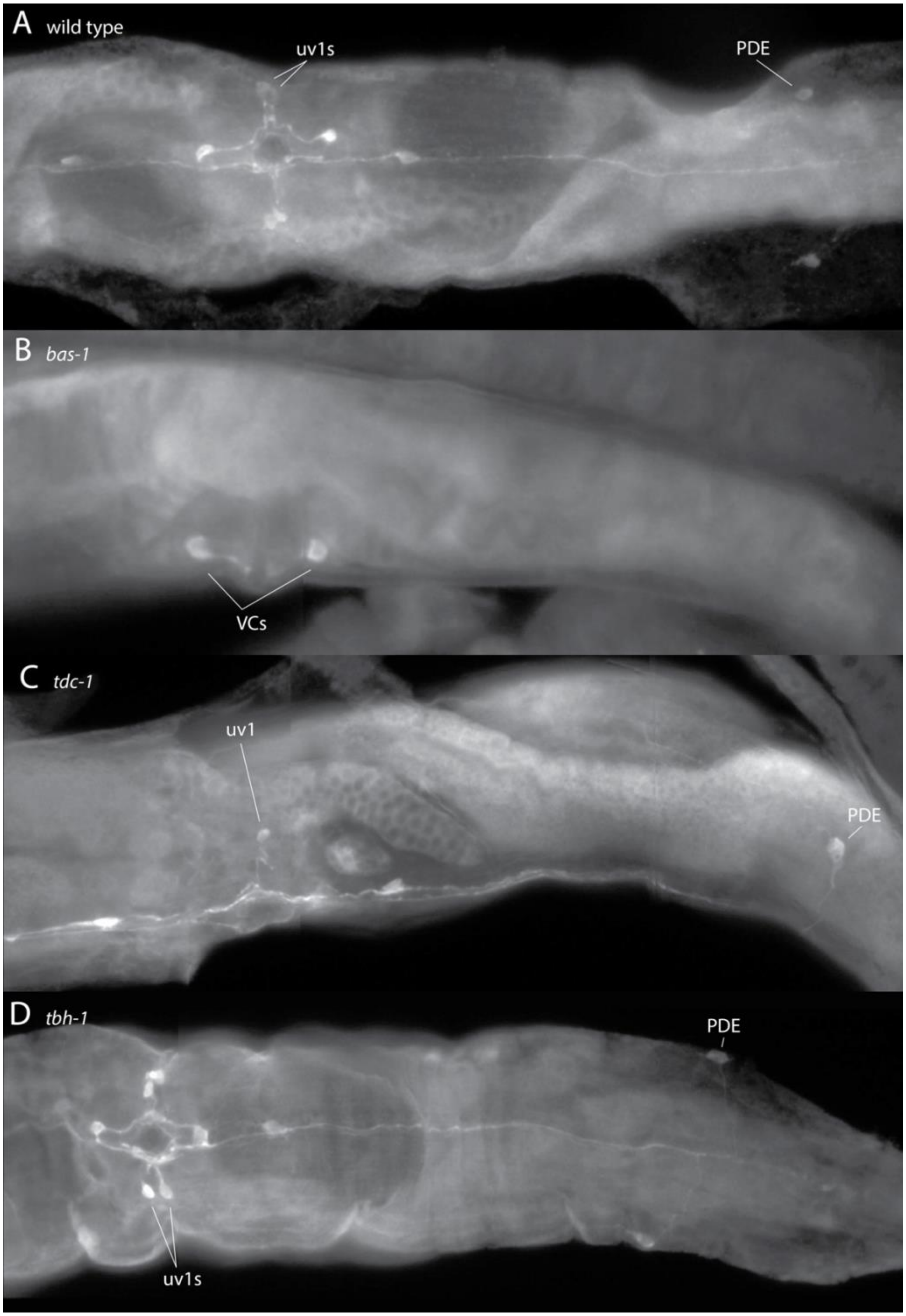

Suppl Fig S14 – 5HTP-induced serotonin immunoreactivity requires *bas-1* / AAADC function
Anterior is to the left, each panel shows anti-serotonin staining in adult hermaphrodite central and posterior body in worms exposed to 5mM 5-HTP in NGM plates for 24 hr. Such treatment typically results in high background staining in nearly all tissues. (A) Wild type (PS312) worms show both serotonin-IR uv1s and PDEs. Ventral view (uv1s and PDEs seen on both sides). (B) *bas-1(tu629)* mutant worms show no stained uv1s or PDEs, showing the requirement for AAADC function to decarboxylate 5HTP to 5HT. Interestingly, *bas-1* mutants show weak to moderately-stained VC neurons and sometimes NSM neurons in the head, independent of 5HTP exposure (not shown). Lateral view. (C) *tdc-1(tu1007)* worms show staining in uv1s and PDEs. Note that in some worms (unrelated to 5HTP exposure), as shown here, only a single uv1 cell is found on a given side. Lateral view. (D) *tbh-1(cbh32)* worms show staining in uv1s and PDEs. Ventral view (uv1s and PDEs seen on both sides).

bioRxiv preprint doi: https://doi.org/10.1101/2025.10.16.682888; this version posted October 16, 2025. The copyright holder for this preprint (which was not certified by peer review) is the author/funder, who has granted bioRxiv a license to display the preprint in perpetuity. It is made available under aCC-BY 4.0 International license.

Suppl Fig S15

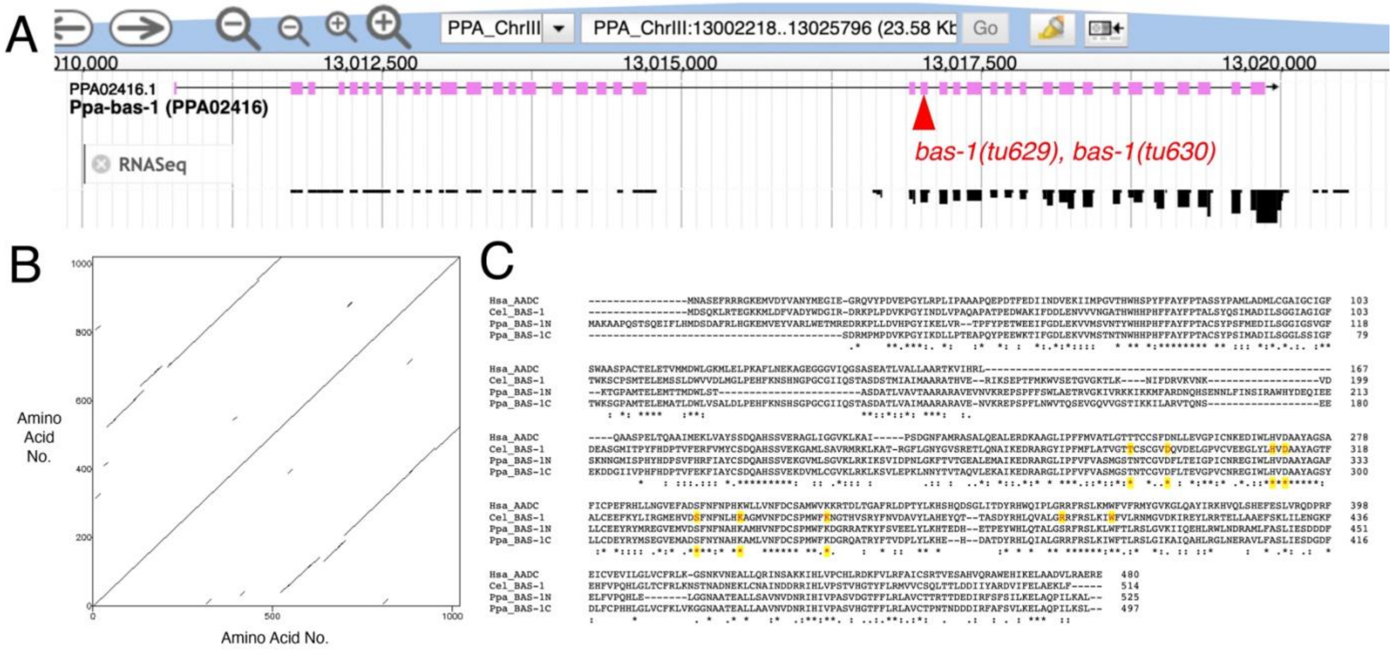

Suppl Fig 15 – *Ppa bas-1* locus is a tandem duplication – *bas-1* mutants may have residual function
(A) *Ppa-bas-1* locus has a tandem duplication of BAS-1/AADC coding sequence (tracks from Wormbase genome browser, WS297). Gene model showing coding exons (pink boxes, upper track) and RNASeq coverage (lower track); existing *bas-1* mutations (causing premature stops) are in the second section of coding sequence (C-terminal duplicate) indicated by red triangle. Differences in RNASeq coverage suggest that the regions are expressed independently. (B) Dot plot of 1022 AA predicted protein (made with https://www.bioinformatics.nl/cgi-bin/emboss/dotmatcher) showing duplicated region starting around 500 AAs. (C) Multiple sequence alignment of human AADC, *C. elegans* BAS-1, *Ppa* N-terminal BAS-1 and C-terminal BAS-1 proteins. Examples of known critical function AAs are highlighted (red letters with yellow background); all but one are conserved in the N-terminal BAS-1 which appears likely to be functional, either by itself or as a truncated internally duplicated protein.

Suppl Fig S16

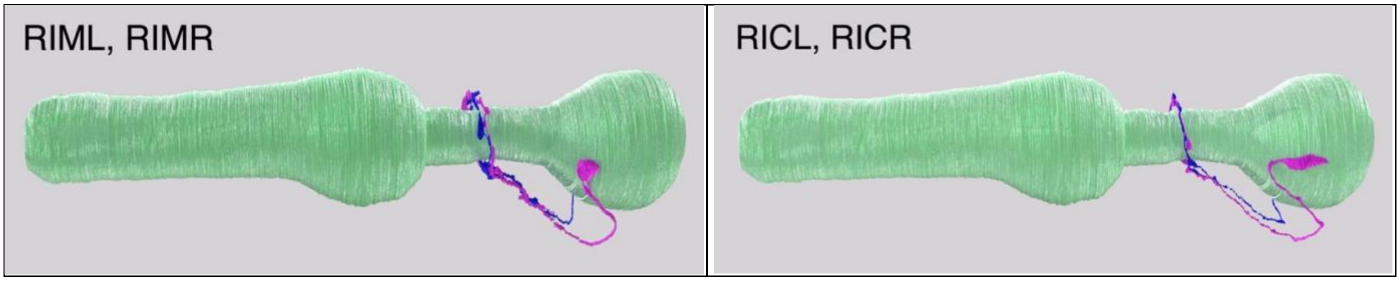

Suppl Fig S16 – *P. pacificus* RIM and RIC neuron 3D renderings
Putative tyraminergic (RIM) and octopaminergic (RIC) neurons, lateral views. Anterior to the left. Pharynx in light green, left neuron in magenta, right side blue (Cook et al., 2025).

bioRxiv preprint doi: https://doi.org/10.1101/2025.10.16.682888; this version posted October 16, 2025. The copyright holder for this preprint (which was not certified by peer review) is the author/funder, who has granted bioRxiv a license to display the preprint in perpetuity. It is made available under aCC-BY 4.0 International license.

Suppl Fig S17 – *Colocalization of cat-1* transcripts with *tdc-1* and *tbh-1* transcripts in RIM and RIC neurons

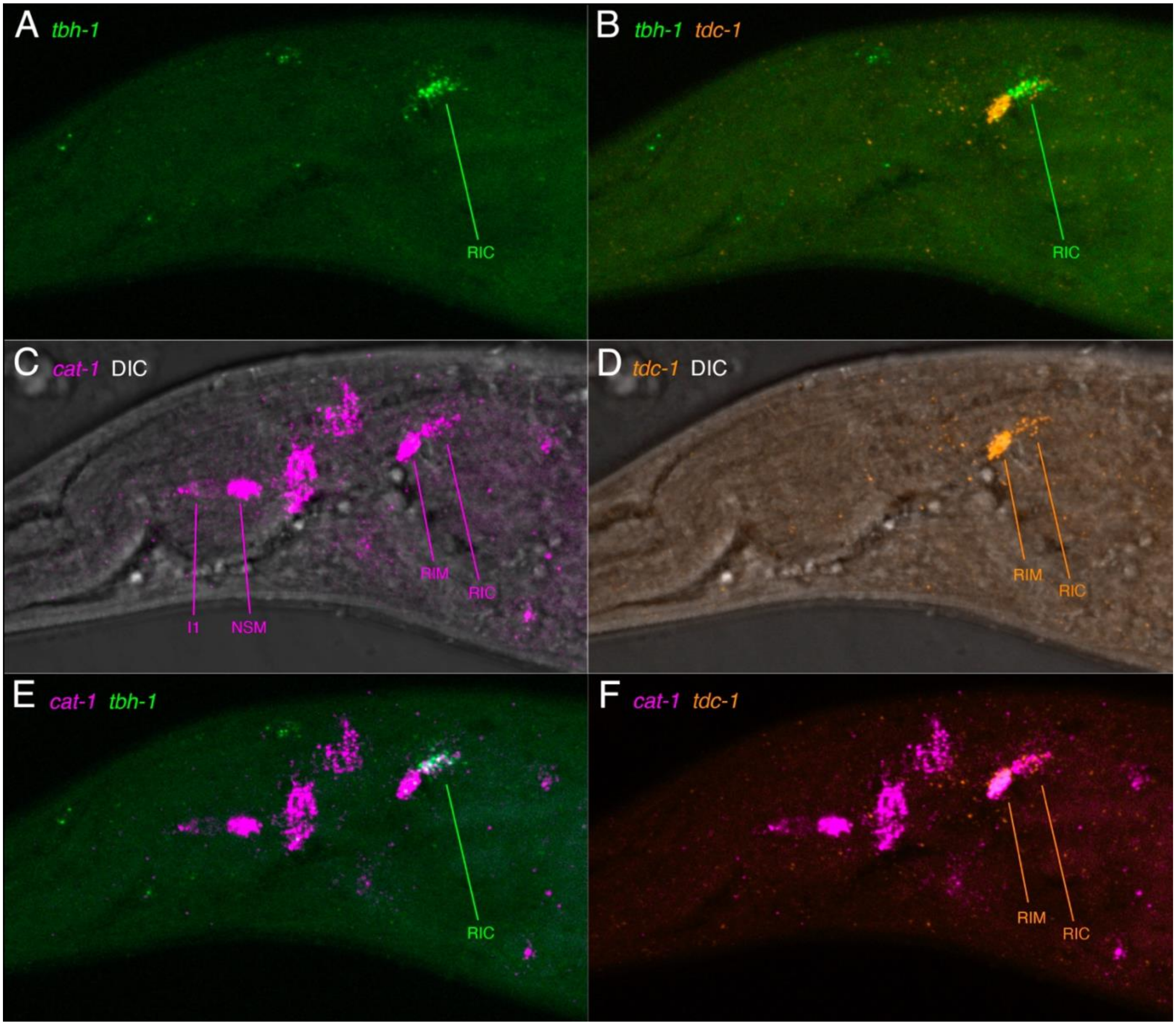

Suppl Fig S17 – *Colocalization of cat-1* transcripts with *tdc-1* and *tbh-1* transcripts in RIM and RIC neurons. Anterior left, ventral down, ventro-lateral view. All images of the same adult hermaphrodite head, MaxIP of a few focal planes showing one side. (A) *tbh-1* transcripts (green) expressed in RIC. (B) *tdc-1* transcripts (orange) , showing two cells in lateral ganglion, RIM and RIC. (C) *cat-1* transcripts (magenta) with DIC to show location of the pharynx as a landmark. Some *cat-1*-positive cells are identified. (D) *tdc-1* with DIC, expression in RIM and RIC. (E) Colocalization of *cat-1* with *tbh-1* transcripts in RIC. (F) Colocalization of *cat-1* with *tdc-1* transcripts in RIM and RIC.

bioRxiv preprint doi: https://doi.org/10.1101/2025.10.16.682888; this version posted October 16, 2025. The copyright holder for this preprint (which was not certified by peer review) is the author/funder, who has granted bioRxiv a license to display the preprint in perpetuity. It is made available under aCC-BY 4.0 International license.

Suppl Fig S18 – Expression of *tbh-1* transcripts in the gonad

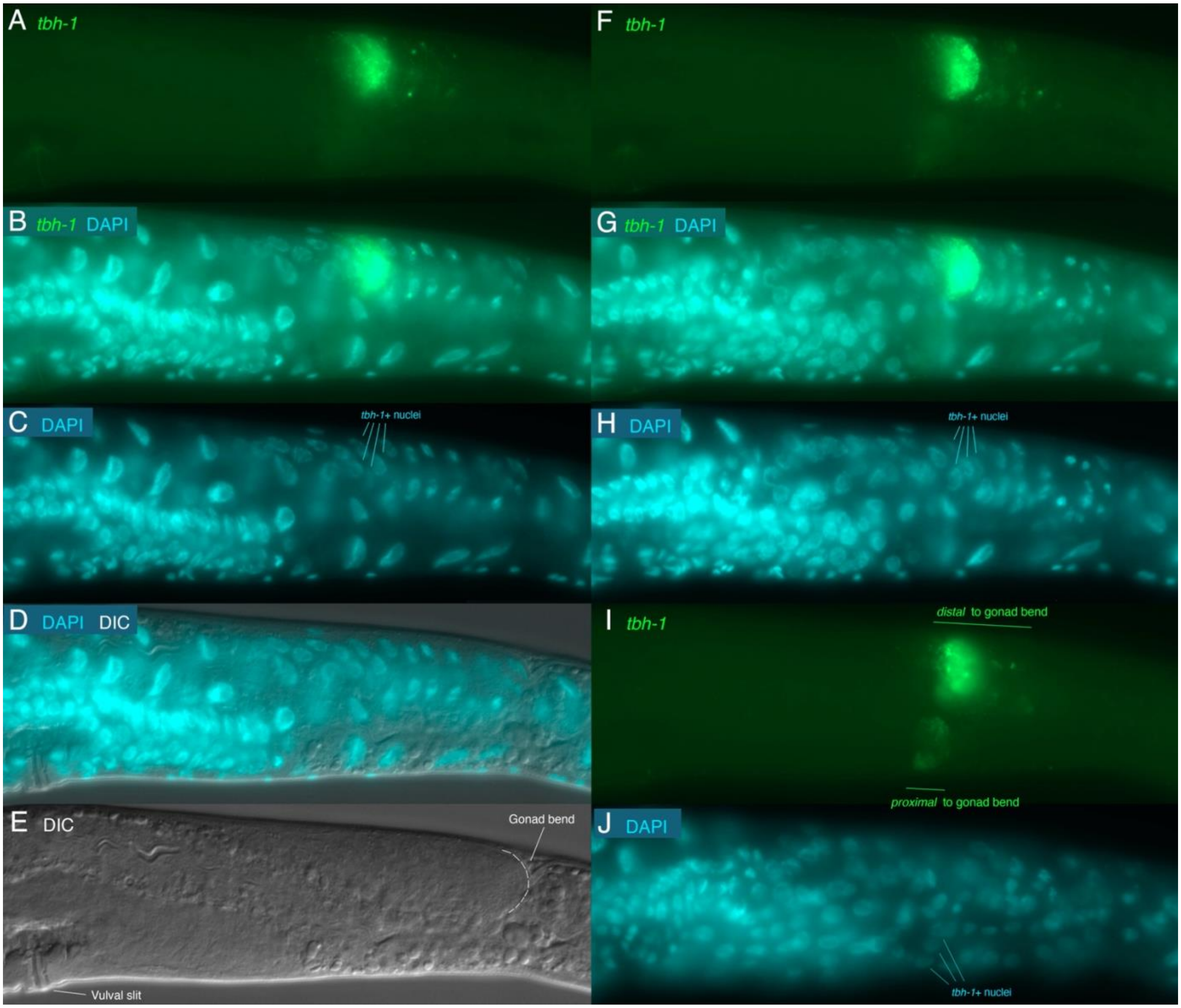

Suppl Fig S18 – Expression of *tbh-1* transcripts in the gonad. Anterior to the left, ventral down, showing posterior body. (A-H) distal (and dorsal) gonad staining ; (A-E) same single focal plane; (F-H) different focal plane. (I, J) same single focal plane, showing proximal (ventral) gonad. 'Proximal' indicates closer to the vulva, distal is further away within the gonad. Regions identified based on (Rudel et al. 2005). (A, F) HCR of *tbh-1* transcripts (green) shows expression in likely gonadal sheath cells. (B, G) Both *tbh-1* and DAPI signals. (C, H) DAPI staining with nuclei strongly *tbh-1*-expressing indicated. Some adjacent nuclei (in front and behind) appear to express *tbh-1* at a lower level. (D) DAPI and DIC. (E) DIC showing that the region of *tbh-1* expression is distal to the gonad bend where the gonad shifts from dorsal to ventral. (I) *tbh-1* transcripts are expressed at a lower level in a second region after the gonad bend (proximal to the vulva). (J) DAPI staining with some *tbh-1*-expressing nuclei indicated.

bioRxiv preprint doi: https://doi.org/10.1101/2025.10.16.682888; this version posted October 16, 2025. The copyright holder for this preprint (which was not certified by peer review) is the author/funder, who has granted bioRxiv a license to display the preprint in perpetuity. It is made available under aCC-BY 4.0 International license.

Suppl Fig S19 – Expression of *tbh-1* transcripts in the proximal gonad is in the spermatheca.

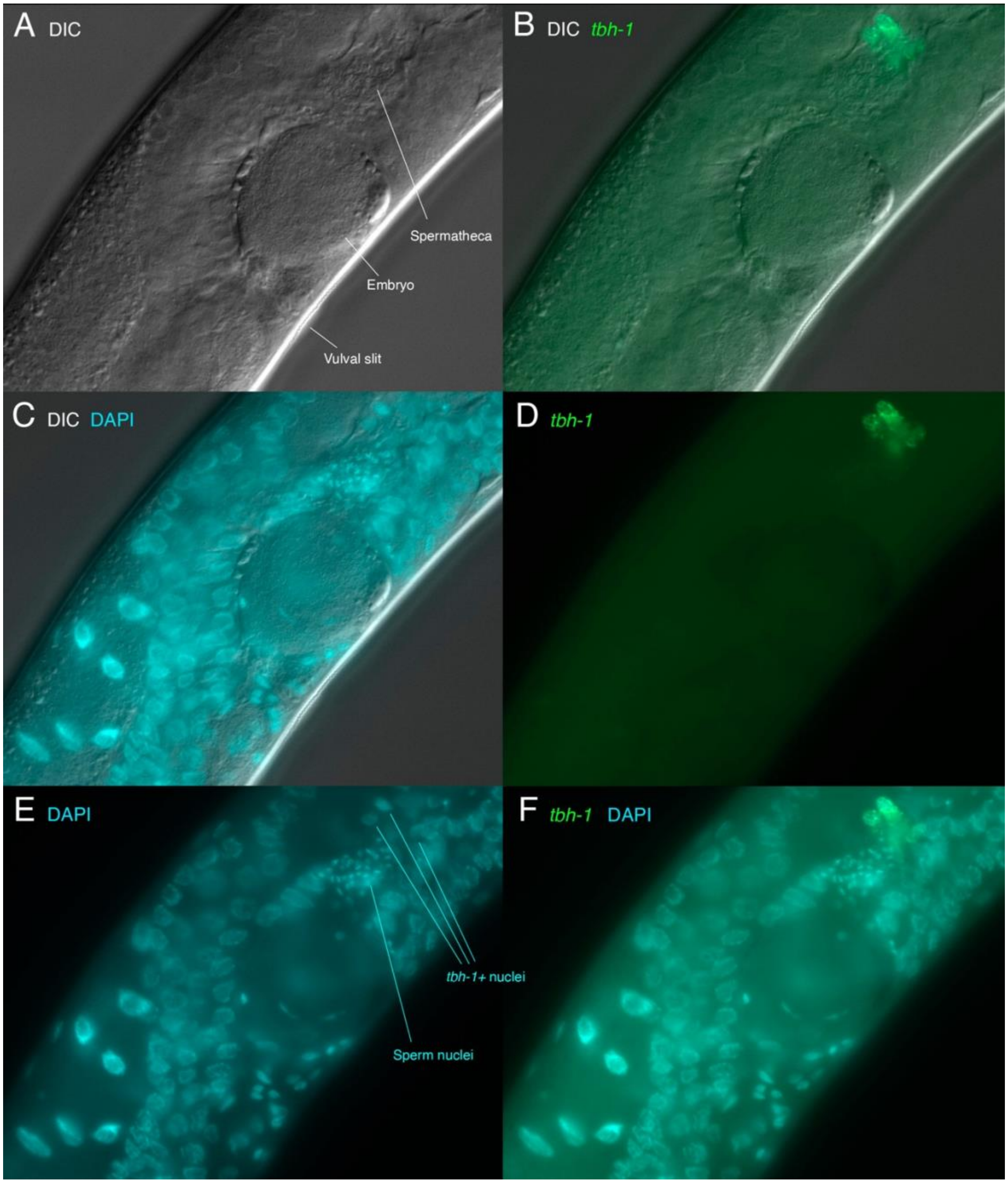

Suppl Fig S19 – Expression of *tbh-1* transcripts in the spermatheca (proximal gonad).
Adult hermaphrodite with embryos, vulval region, dorsal to the upper left, ventral is lower right. Same focal plane for each panel. (A) DIC showing locations as indicated. A single embryo is present in the uterus between the vulval opening and more distal spermatheca. (B, D, F) Expression of *tbh-1* transcripts by HCR. (B) *tbh-1* transcripts (green) + DIC. (C) DIC and DAPI (cyan) to show nuclei. (D) *tbh-1* alone. (E) DAPI with sperm nuclei within spermatheca indicated. Just distally is gonad constriction with *tbh-1*-expressing nuclei indicated. (F) *tbh-1* transcripts + DAPI staining.

bioRxiv preprint doi: https://doi.org/10.1101/2025.10.16.682888; this version posted October 16, 2025. The copyright holder for this preprint (which was not certified by peer review) is the author/funder, who has granted bioRxiv a license to display the preprint in perpetuity. It is made available under aCC-BY 4.0 International license.

Suppl Fig S20 – Expression of *cat-1* and *tdc-1* transcripts in adjacent vulval cells

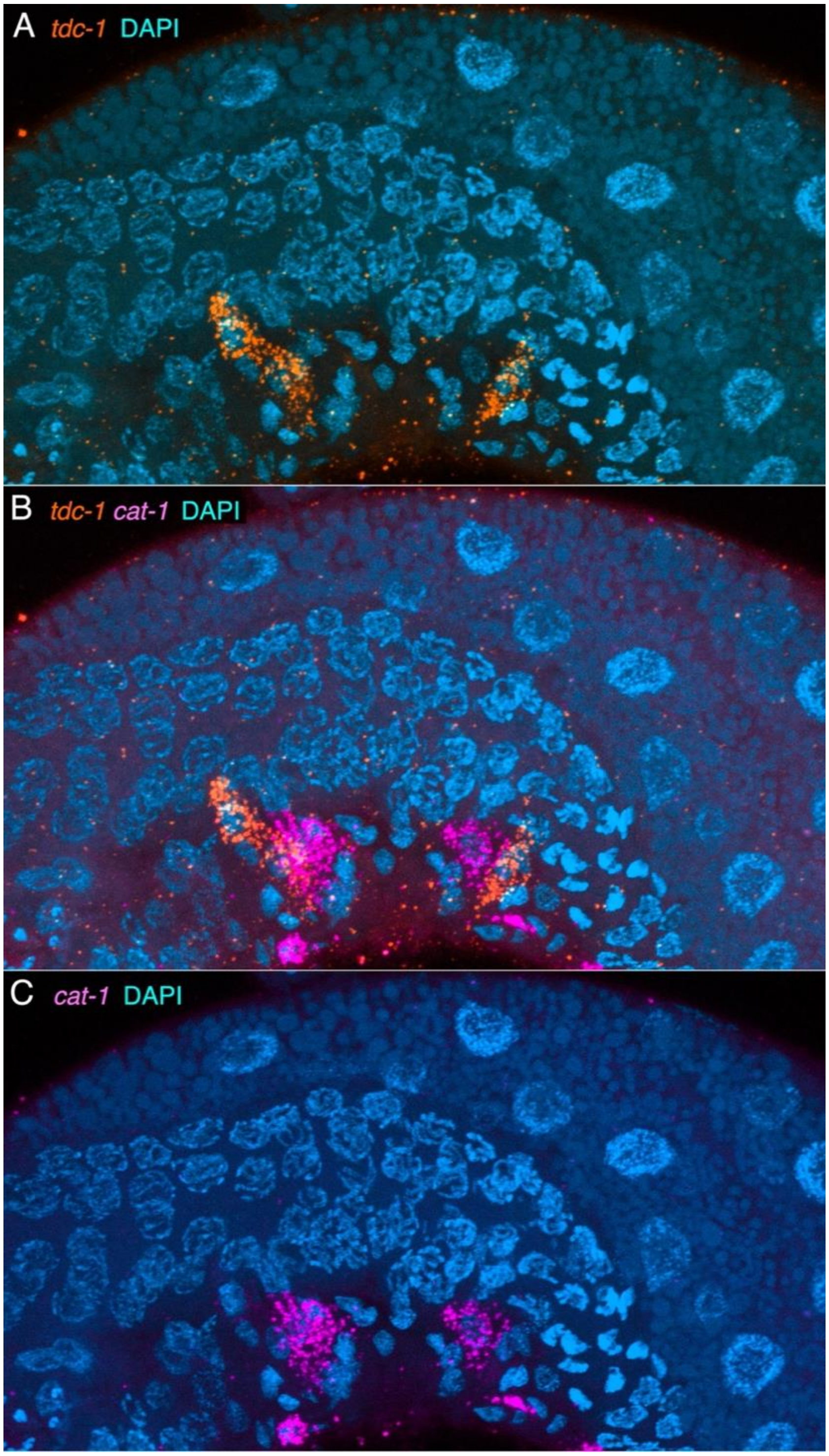

Suppl Fig S20. Expression of *tdc-1* and *cat-1* transcripts is in adjacent vulval region cells. Adult hermaphrodite vulval region, anterior to the right and ventral down, MaxIP of 21 z-planes on the left side to approximately the midline. (A) *tdc-1* transcripts (orange) associated with two nuclei (DAPI, cyan) per quandrant. (B) Co-labeling shows that *cat-1* transcripts (magenta) are associated with two nuclei closer to the vulval pore in each quadrant. Although this image suggests possible overlap in expression in some cells (left / anterior), most preparations show clear separation of expression in adjacent cells, like that seen here in the posterior cells. (C) *cat-1* transcripts expressed close to the vulval pore, lateral to the ventral nerve cord. Some smaller VC neurons expressing *cat-1* are seen at the bottom of the image.

bioRxiv preprint doi: https://doi.org/10.1101/2025.10.16.682888; this version posted October 16, 2025. The copyright holder for this preprint (which was not certified by peer review) is the author/funder, who has granted bioRxiv a license to display the preprint in perpetuity. It is made available under aCC-BY 4.0 International license.

Suppl Fig S21

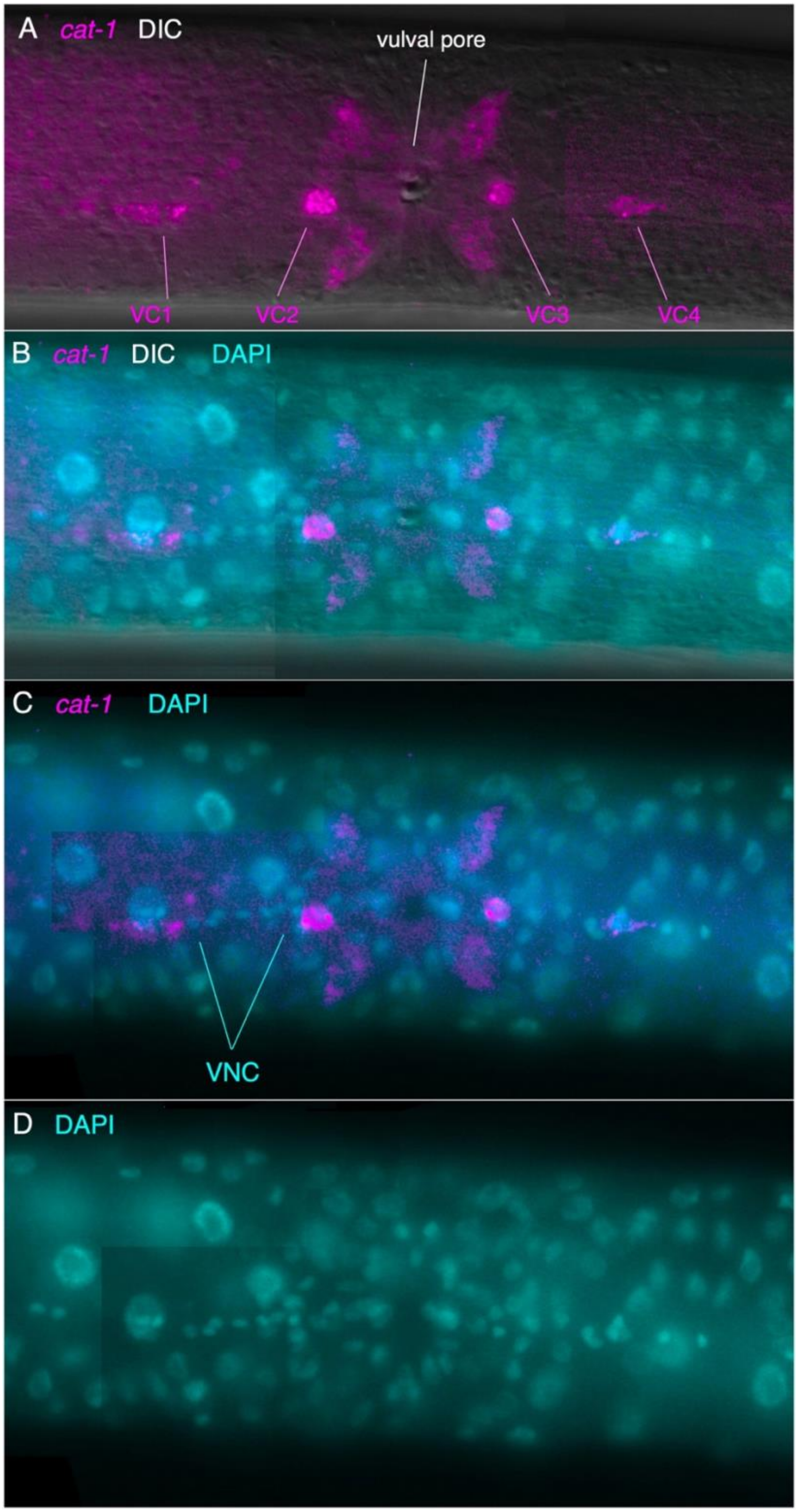

Suppl Fig S21 – Expression of *cat-1* transcripts in the midbody VC neurons and vulval cells. Ventral view; anterior is to the left in all images. Each panel is the same single focal plane (panels B-D, however, a partly montages that include a small region from a nearby focal plane to better show the ventral nerve cord). (A-C) HCR for *cat-1* transcripts (magenta) with DIC and/or DAPI (cyan). (D) DAPI alone to better show vulval region and VNC.

bioRxiv preprint doi: https://doi.org/10.1101/2025.10.16.682888; this version posted October 16, 2025. The copyright holder for this preprint (which was not certified by peer review) is the author/funder, who has granted bioRxiv a license to display the preprint in perpetuity. It is made available under aCC-BY 4.0 International license.

Suppl Fig S22 – Other *cat-1*-expressing cells in the head.

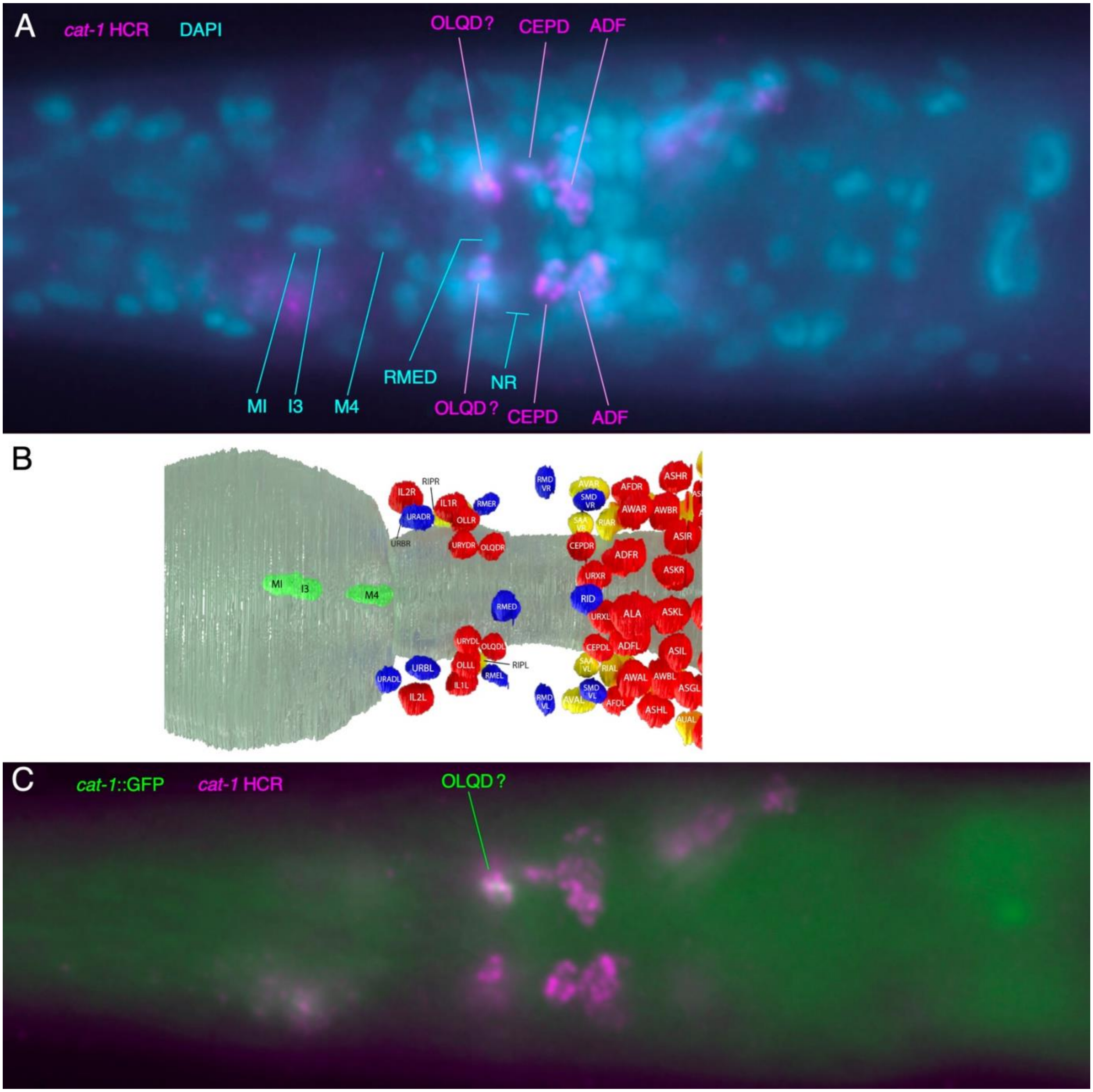

Suppl Fig S22 – Other *cat-1*-expressing cells in the head. Expression of *cat-1* transcripts via HCR in dorsal head in *cat-1*::GFP strain. Anterior to the left. (A, C) same dorsal focal plane. (A) *cat-1* HCR fluorescence and DAPI, particularly showing likely identification of OLQDs as dorsal anterior ganglion nuclei just anterior to the nucleus-free region of the nerve ring (NR). Readily identifiable DAPI stained nuclei in the dorsal anterior pharyngeal bulb are indicated. (B) Dorsal view map of dorsal neuronal nuclei around NR, including dorsal pharyngeal neurons (green) identified in (A) from EM reconstruction (Cook et al., 2025); pharynx, partly transparent, in darker green. Other head nuclei (non-pharyngeal) colored by apparent function: sensory (red), motor (blue), interneuron (yellow). (C) *cat-1*::GFP transgene expression and *cat-1* HCR fluorescence colocalization in one putative OLDQ neuron. Same focal plane as (A).

bioRxiv preprint doi: https://doi.org/10.1101/2025.10.16.682888; this version posted October 16, 2025. The copyright holder for this preprint (which was not certified by peer review) is the author/funder, who has granted bioRxiv a license to display the preprint in perpetuity. It is made available under aCC-BY 4.0 International license.

Suppl Fig S23 – *Acrobeloides nanus* ES501 head serotonin-IR neurons

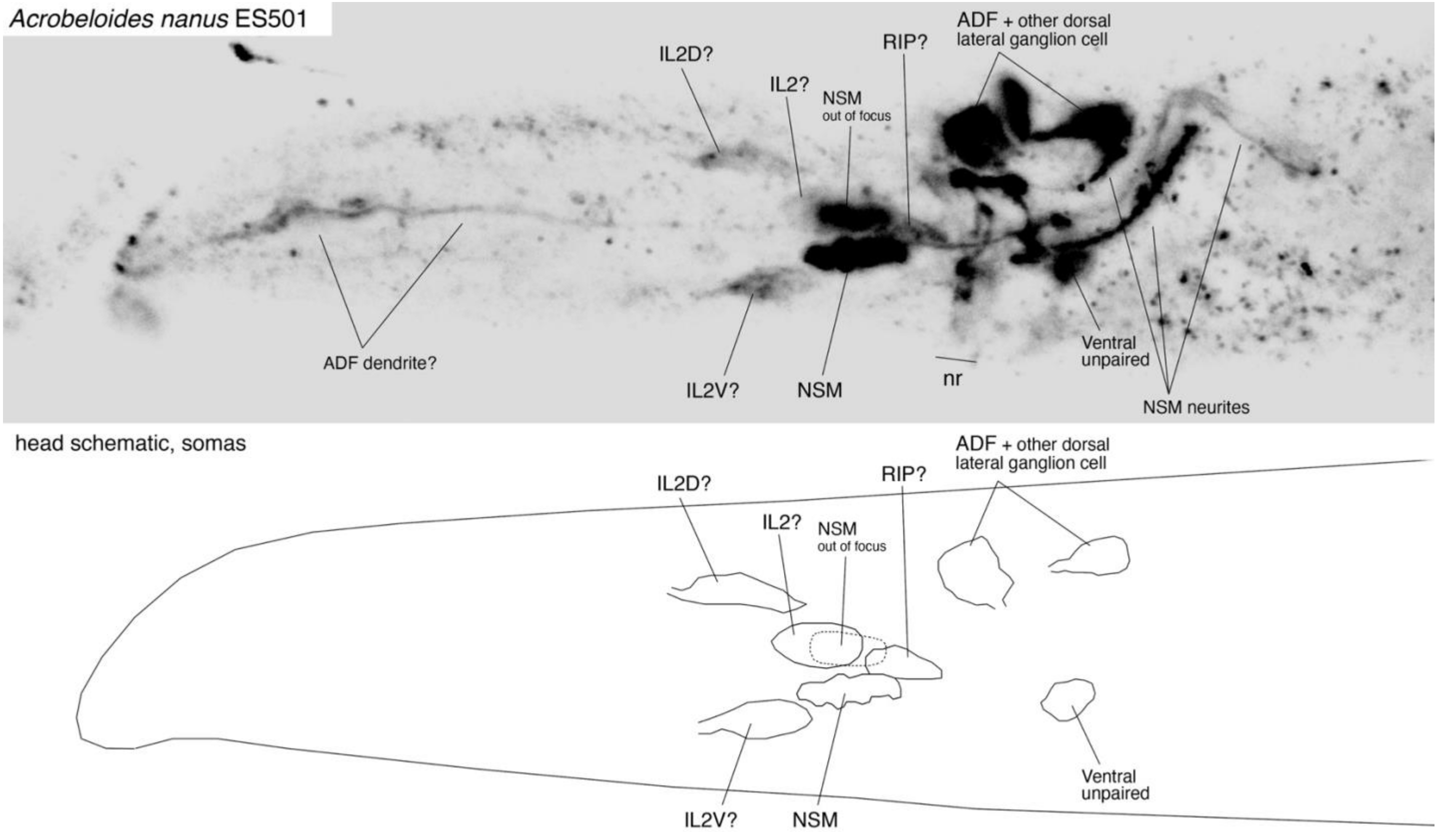

Suppl Fig S23 – *Acrobeloides nanus* ES501 head serotonin-IR neurons. Anterior left, ventral down, lateral view; confocal MaxIP of a few focal planes on left side (top); likely cell and neurite identifications as indicated. Brighter cells and neurites are over-exposed to show less brightly stained somas. nr – location of nerve ring. Schematic showing outlines of somas matching image (below).

bioRxiv preprint doi: https://doi.org/10.1101/2025.10.16.682888; this version posted October 16, 2025. The copyright holder for this preprint (which was not certified by peer review) is the author/funder, who has granted bioRxiv a license to display the preprint in perpetuity. It is made available under aCC-BY 4.0 International license.

Suppl Fig S24 – *Plectus sambesii* ES601 head serotonin-IR neurons

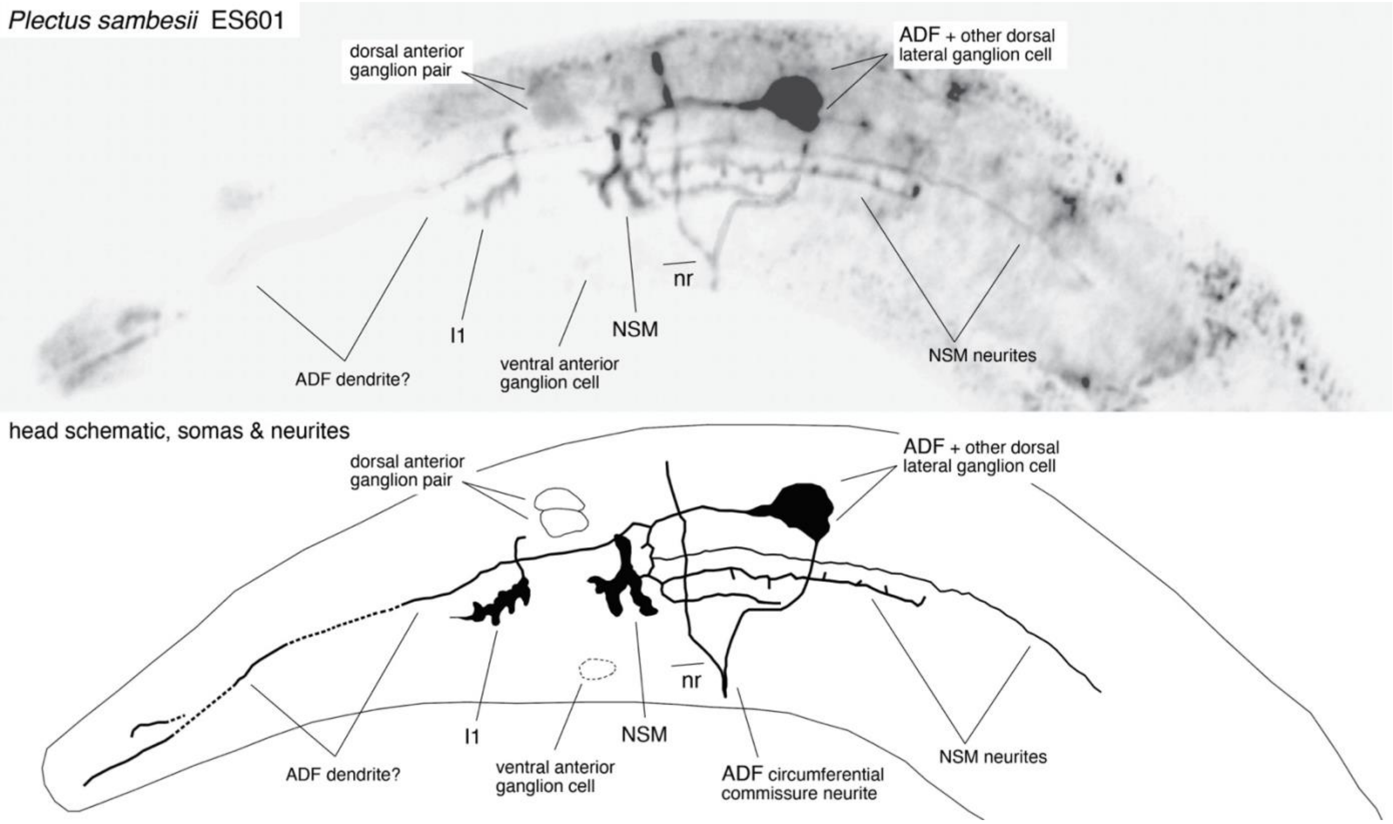

Suppl Fig S24 – *Plectus sambesii* ES601 head serotonin-IR neurons. Anterior left, ventral down, lateral view; montage of confocal focal planes on left side (top); likely cell and neurite identifications as indicated. nr – location of nerve ring. Schematic showing outlines of somas and neurites matching image (below).

bioRxiv preprint doi: https://doi.org/10.1101/2025.10.16.682888; this version posted October 16, 2025. The copyright holder for this preprint (which was not certified by peer review) is the author/funder, who has granted bioRxiv a license to display the preprint in perpetuity. It is made available under aCC-BY 4.0 International license.

Suppl Fig S25 – *Plectus sp.* WWL501 head serotonin-IR neurons.

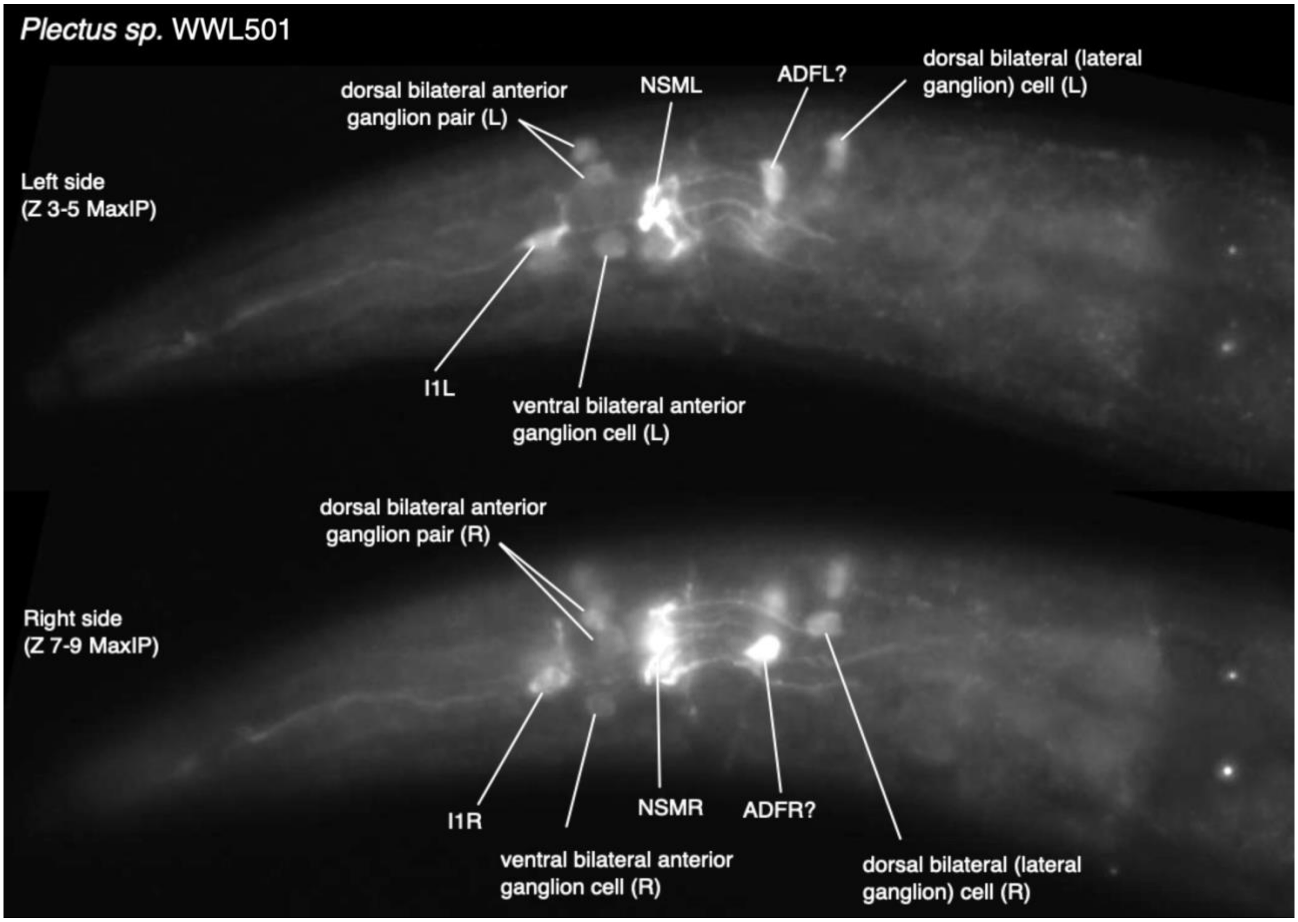

Suppl Fig S25 – *Plectus sp.* WWL501 head serotonin-IR neurons. Anterior left, ventral down, lateral views; confocal MaxIPs of a few focal planes on left (top) and right (bottom) sides. Likely cell identifications as indicated.

Suppl Fig S26A – *P. pacificus* CAT-1 mutant alignments

```
CAT-1(wildtype): MTIAEFIARNRGNRKALLLIVYIALFLDNMLLTTV…
CAT-1(csu115):   MTIAEFIARNRGNRKALLLIVYIALFLDNMLLTTLI*
CAT-1(csu116):   MTIAEFIARNRGNRKALLLIVYIALFLDNMLLTTV*
```

Asterisks indicated premature stop codons in predicted mutant CAT-1 proteins.

Suppl Fig S26B – *P. pacificus* DAT-1 mutants (N-terminal deletions)

Wild type sequence surrounding predicted translation start site and targeted PAM site (green)

```
                                     predicted cut site|   PAM
gattcgatcgatactctcttttgatggccgatgatcagtgATGGACACCGCAGCCCAGGAGCAGGgtgggttcggtttaaaat
                                         M  D  T  A  A  Q  E  Q
                                        Exon 1                    Intron 1
```

dat-1(pa507): a 303 bp deletion surrounding the predicted CRISPR cut site.
dat-1(pa508): a 441 bp deletion/12 bp insertion surrounding the predicted CRISPR cut site.

bioRxiv preprint doi: https://doi.org/10.1101/2025.10.16.682888; this version posted October 16, 2025. The copyright holder for this preprint (which was not certified by peer review) is the author/funder, who has granted bioRxiv a license to display the preprint in perpetuity. It is made available under aCC-BY 4.0 International license.

Suppl Fig 27. Anti-5HT staining in *P. pacificus cat-1* mutants is variably reduced.

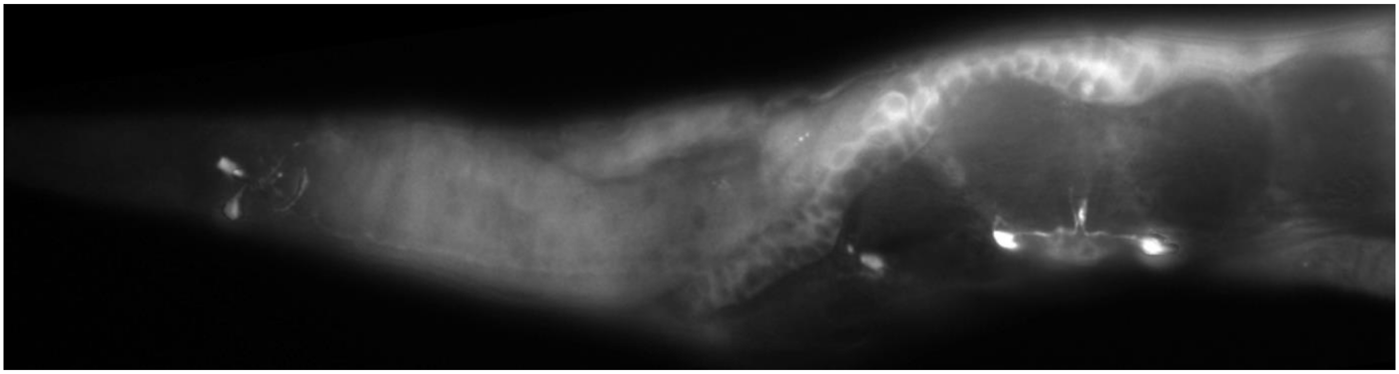

Suppl Fig 27. Anti-5HT staining in *P. pacificus cat-1* mutants is variably reduced. Anterior to the left, ventro-lateral view, montage. Head and midbody VNC region of *cat-1(csu116)* adult hermaphrodite. In this individual, NSM neurons in the head are moderately stained; other serotonin-IR neurons are not seen. VC neurons in VNC stain similarly to wildtype (VC1-3 shown; VC4, which also stains moderately, is out of the plane of focus). Although both *cat-1* mutations likely cause a complete loss of function, we observed a variable reduction in serotonin-IR, as has been seen previously in *C. elegans cat-1* mutants (Desai et al., 1988) (Loer and Kenyon 1993). Remaining serotonin-IR is not surprising since neurons should continue to synthesize serotonin via *tph-1*/TPH and *bas-1*/AADC expression (Figure 1), even if the cells are unable to package the neurotransmitter into synaptic vesicles. In some preparations, we saw loss of serotonin-IR in most or all head neurons and midbody VC neurons; in other preparations (like that above), we observed reduced but considerable staining in all known serotonin-IR cells. The behavioral phenotypes we observed in *P. pacificus cat-1* mutants are consistent with a loss of VMAT function in spite of remaining neurotransmitter within the neurons.

bioRxiv preprint doi: https://doi.org/10.1101/2025.10.16.682888; this version posted October 16, 2025. The copyright holder for this preprint (which was not certified by peer review) is the author/funder, who has granted bioRxiv a license to display the preprint in perpetuity. It is made available under aCC-BY 4.0 International license.

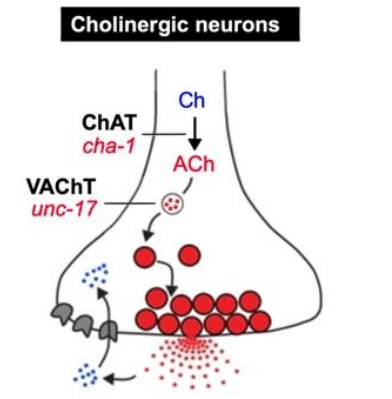

Suppl Fig S28 – Cholinergic proteins and nematode genes
Proteins encoded by identified nematode genes are indicated in black, bold, capital letters (abbreviations: Ch – choline; ACh – acetylcholine; ChAT – choline acetyltransferase, required for acetylcholine synthesis; VAChT – vesicular acetylcholine transporter, required for packaging acetylcholine into synaptic vesicles); nematode gene names (originally from *C. elegans*) are italicized below the protein name abbreviations.

Suppl Fig S29 – Cholinergic neurons and nerves in the head of *P. pacificus*

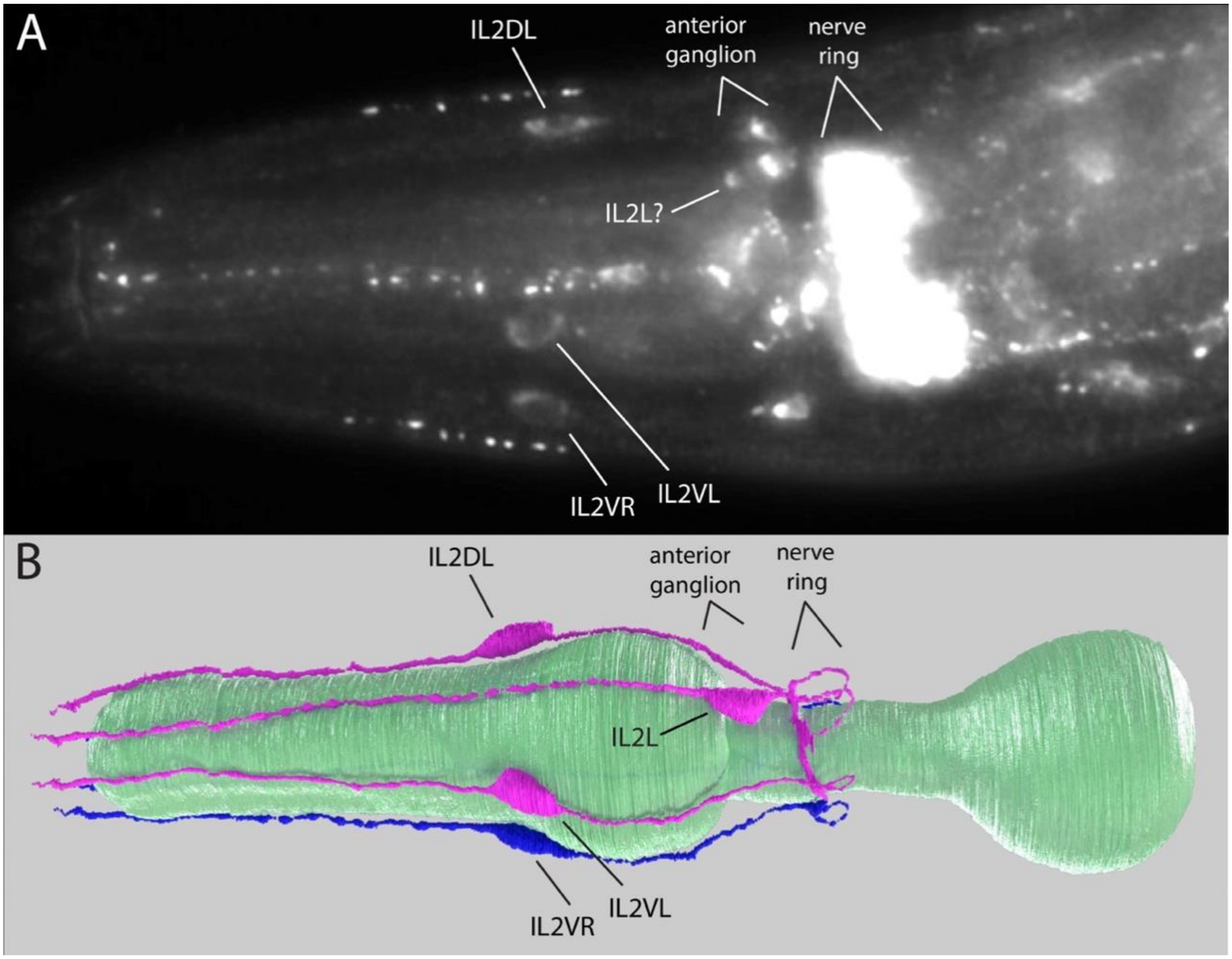

Suppl Fig S29 – Cholinergic neurons and nerves in the head of *P. pacificus*
Both images are left side views, anterior to the left. (A) Wildtype adult hermaphrodite head staining with a 'cholinergic mix' of monoclonal antibodies to *C. elegans* cholinergic proteins CHA-1 (ChAT) and UNC-17 (VAChT) shows extensive staining in the nervous system. The nerve ring is very strongly stained; head ganglia surrounding the nerve ring, including the anterior ganglion, are moderately stained. Although it is difficult to identify individual neuronal somas, the somas of IL2D and IL2V neurons are well isolated from all other neurons, being just in front of the pharyngeal anterior bulb, so can be clearly identified. In this view, the IL2DL (left) is seen, and both IL2Vs (L & R). Another soma in the anterior ganglion could be IL2L. (B) 3D rendering of IL2 neurons, left lateral view (and slightly ventral), anterior to the left. IL2Ds and IL2Vs are further anterior; IL2 L & R are within the anterior ganglion near to the nerve ring. Left side IL2s are rendered in magenta; right side IL2s in blue. The pharynx is in green.

bioRxiv preprint doi: https://doi.org/10.1101/2025.10.16.682888; this version posted October 16, 2025. The copyright holder for this preprint (which was not certified by peer review) is the author/funder, who has granted bioRxiv a license to display the preprint in perpetuity. It is made available under aCC-BY 4.0 International license.

Suppl Fig S30 – CHA-1 C-terminus alignments of mutant alleles and wildtype proteins.

A

```
CHA-1(wildtype):…RDALREMKSLLDRE*
CHA-1(ot5000):  …RDALREMKSLLDYKDDDDKDYKDDDDKDRE* (2x FLAG)
CHA-1(ot5001):  …RDALREMKSLYRNPPQCELQCELM*
CHA-1(ot5002):  …HTXTSLAGFKNAIRDALREMKSEKHRNLRI*
```

B

```
Cel-CHA-1   568   DCYLTYGAVVRDGYGCPYNIQPDRVIFAPTAFRSDPRTDLQHFKKSLAGAMRDVKELLSN*   627
                    YL YGAVVRDGYGC YNI P+R+IFAP+AFRS+ RT L  FK ++  A+R++K LL
Ppa-CHA-1   570   GSYLCYGAVVRDGYGCAYNIMPNRIIFAPSAFRSNTRTSLAGFKNAIRDALREMKSLLDRE*  630
                    + CYG VV +GYG  YN  P  I+F  S+F S   TS + F  A+ ++L +M+ L
Hsa-ChAT    664   --FCCYGPVVPNGYGACYNPQPETILFCISSFHSCKETSSSKFAKAVEESLIDMRDLCSLL.. 725 (748 total)
                                                              Helix 20
```

Suppl Fig S30 – CHA-1 C-terminus alignments of mutant alleles and wildtype proteins.

(A) Alignments of wildtype *P. pacificus* CHA-1 protein showing alterations in mutant alleles. * denotes stop codon; **bolded** region shows in-frame 2x FLAG epitope tag insertion (DYKDDDDK); underlined regions indicate results of frame-shift from indel mutations.

(B) Alignments of C-termini from nematode and mammalian proteins. There is considerable conservation of the C-terminus. Between each sequence shows identical and similar (+) AAs. *Cel – C. elegans, Ppa – P. pacificus, Hsa – Homo sapiens.* *Cel* sequence highlight: P584L missense mutation results in 99% loss of ChAT activity (Rand and Russel, 1984). *Ppa* sequence highlight: *Ppa* CHA-1 wildtype C-terminus portion shown in *Ppa* mutant alignments (A). *Hsa*-ChAT highlight: region of Helix 20 in rat ChAT crystal structure (Govindasamy et al., 2004).

bioRxiv preprint doi: https://doi.org/10.1101/2025.10.16.682888; this version posted October 16, 2025. The copyright holder for this preprint (which was not certified by peer review) is the author/funder, who has granted bioRxiv a license to display the preprint in perpetuity. It is made available under aCC-BY 4.0 International license.

Suppl Fig S31

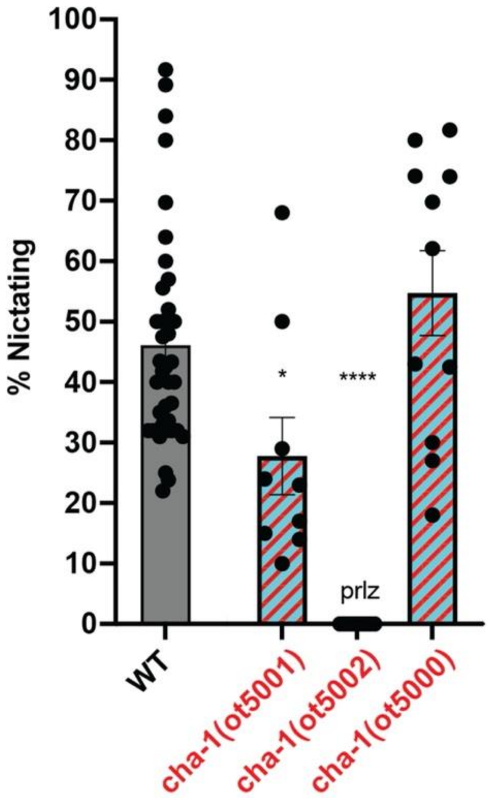

Suppl Fig S31 – Effect of cholinergic function loss on nictation is inconclusive
Reduction-of-function alleles in the acetylcholine biosynthesis gene *cha-1*, including the C-terminal 2x-FLAG strain (*ot5000*), and two C-terminal indel mutants were found in the same CRISPR knock-in screen (*ot5001, ot5002*) (Suppl Figure 19) have temperature-sensitive phenotypes.† In these nictation assays, the alleles showed a partial nictation phenotype (*ot5001*), paralysis of locomotion (*ot5002*), or no effect (*ot5000,* in frame FLAG insertion). Between 30-60 animals participate in each nictation assay and at least 6 assays were performed for each genotype. *P<0.05, ***P<0.001, ****P<0.0001 Dunnett's multiple comparisons test show significant difference to wildtype (WT).

† All three *cha-1* strains were found to be temperature-sensitive; this may reflect a temperature-sensitive process in CHA-1 protein function such has been observed in all *C. elegans cha-1* mutants (Duerr et al., 2021). Whereas the mutants appeared to move similarly to wildtype when raised at 20°C, mutants raised continuously at 25°C or higher became extremely uncoordinated or paralyzed, and progressively become sterile, particularly at temperatures above 25°C. Therefore, to test *cha-1* dauers, we let cultures starve at the permissive 20°C, then shifted to 25°C after food was depleted but before dauer formation, so that dauers were formed at the higher temperature. Given the pleiotropic phenotype of the *cha-1* alleles at 25ºC, however, including defects in locomotion, whether the phenotype is truly specific to nictation is inconclusive. Furthermore, the nictation phenotype could also result simply from a slower response to the sand substrate beyond the 30-minute incubation time of the assay.
